# A Cold-Active Flavin-Dependent Monooxygenase from *Janthinobacterium svalbardensis* Unlocks Applications of Baeyer–Villiger Monooxygenases at Low Temperature

**DOI:** 10.1021/acscatal.2c05160

**Published:** 2023-02-27

**Authors:** Andrea M. Chánique, Nakia Polidori, Lucija Sovic, Daniel Kracher, Leen Assil-Companioni, Philipp Galuska, Loreto P. Parra, Karl Gruber, Robert Kourist

**Affiliations:** † NAWI Graz, BioTechMed-Graz, Institute of Molecular Biotechnology, Graz University of Technology, Petersgasse 14, Graz 8010, Austria; ‡ Department of Chemical and Bioprocesses Engineering, School of Engineering, Pontificia Universidad Católica de Chile, Vicuña Mackenna 4860, Santiago 7810000, Chile; § NAWI Graz, BioTechMed Graz, Institute of Molecular Biosciences, University of Graz, Humboldtstraße 50, Graz 8010, Austria; ∥ ACIB GmbH, Petersgasse 14/1, Graz 8010, Austria; ⊥ Schools of Engineering, Medicine and Biological Sciences, Institute for Biological and Medical Engineering, Pontificia Universidad Católica de Chile, Vicuña Mackenna 4860, Santiago 7810000, Chile

**Keywords:** biocatalysis, enantioselectivity, cold-active, Baeyer−Villiger monooxygenase, cofactor promiscuity

## Abstract

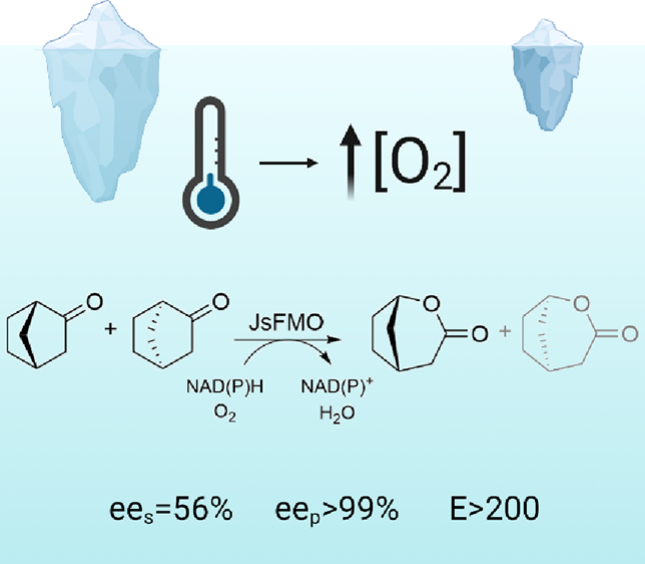

Cold-active enzymes maintain a large part of their optimal activity at low temperatures. Therefore, they can be used to avoid side reactions and preserve heat-sensitive compounds. Baeyer–Villiger monooxygenases (BVMO) utilize molecular oxygen as a co-substrate to catalyze reactions widely employed for steroid, agrochemical, antibiotic, and pheromone production. Oxygen has been described as the rate-limiting factor for some BVMO applications, thereby hindering their efficient utilization. Considering that oxygen solubility in water increases by 40% when the temperature is decreased from 30 to 10 °C, we set out to identify and characterize a cold-active BVMO. Using genome mining in the Antarctic organism *Janthinobacterium svalbardensis,* a cold-active type II flavin-dependent monooxygenase (FMO) was discovered. The enzyme shows promiscuity toward NADH and NADPH and high activity between 5 and 25 °C. The enzyme catalyzes the monooxygenation and sulfoxidation of a wide range of ketones and thioesters. The high enantioselectivity in the oxidation of norcamphor (eeS = 56%, eeP > 99%, *E* > 200) demonstrates that the generally higher flexibility observed in the active sites of cold-active enzymes, which compensates for the lower motion at cold temperatures, does not necessarily reduce the selectivity of these enzymes. To gain a better understanding of the unique mechanistic features of type II FMOs, we determined the structure of the dimeric enzyme at 2.5 Å resolution. While the unusual N-terminal domain has been related to the catalytic properties of type II FMOs, the structure shows a SnoaL-like N-terminal domain that is not interacting directly with the active site. The active site of the enzyme is accessible only through a tunnel, with Tyr-458, Asp-217, and His-216 as catalytic residues, a combination not observed before in FMOs and BVMOs.

## 1 Introduction

The complexity and variety of extreme conditions on our planet have led to various adaptations of lifeforms. As a result, we find organisms with rare properties that can potentially be exploited for industrial processes. Cold-adapted microorganisms, which exist in polar regions, deep ocean waters, marine sediments, permafrost, and high mountains, constitute such organisms.^1^ As an adaptation to their cold environment, they utilize enzymes that have evolved to catalyze reactions at low temperatures.^2^ While these cold-active enzymes exhibit a significant fraction of their activity at low temperatures, most exhibit optimal temperatures in the mesophilic range between 25 and 40 °C.^1^ Cold-active enzymes are adapted to cope with the reduced chemical rates of reactions at low temperatures while maintaining their stability, avoiding cold-induced unfolding, and keeping the dynamics of their structures.^1^ They accomplish this by increasing their flexibility in regions close to the active site compared to mesophilic enzymes at the same temperature and reducing the number of enzyme–ligand interactions that must be broken during the reaction.^3^

The high flexibility and the low number of interactions of the active site widen the substrate spectra of cold-active enzymes compared to their mesophilic counterparts.^4^ This property is useful for industrial processes, where it is possible to use the same biocatalyst for several substrates of interest with similar molecular structures. Enzymes from psychrophilic organisms also have a tighter hydration shell at a given temperature,^5^ making them more tolerant to organic solvent exposure.^6^ Another interesting aspect of cold-active enzymes is that they can be easily inactivated by exposure to heat after they are used. Activity at low to medium temperatures implies that cold-active enzymes can be utilized at room temperature, eliminating the need for heating and, therefore, lowering energy consumption. Their high activity at low temperatures can also be exploited to conduct reactions at temperatures below 10 °C as a means to avoid undesirable side reactions—as most enzymes from mesophilic production hosts are not active at this low range.^1^ Cold activity is also practical when working with thermosensitive compounds, especially in the food industry and for molecular biology applications.^7^ Another interesting application is their use in biphasic systems, as operating at low temperatures facilitates the diffusion of the products from one phase to the other,^8^ a feature that is particularly attractive for enzymes suffering from substrate or product inhibition. Furthermore, gaseous co-substrates such as oxygen often exhibit higher solubilities in water at lower temperatures, making cold activity particularly attractive for enzymatic reactions requiring such co-substrates.^9^ In the particular case of oxygen, solubility in water at 10 °C is 40% higher than at 30 °C.^10^

Baeyer–Villiger monooxygenations are reactions widely used in organic synthesis. Examples include the preparation of steroids, agrochemicals, antibiotics, and pheromones.^11,12^ The reaction introduces an oxygen atom adjacent to a carbonyl group, generating the corresponding ester or lactone.^13^ Recently, the production of trimethyl-ε-caprolactone from 3,3,5-trimethyl-cyclohexanone was performed at the 100 L scale using a BVMO from *Thermocrispum municipale,*(14) demonstrating the industrial potential of these enzymes. Baeyer–Villiger monooxygenases (BVMOs) are flavin-containing enzymes capable of catalyzing this reaction utilizing the nicotinamide cofactors NADH or NADPH and oxygen as a co-substrate, generating water as a byproduct.^12^ BVMOs operate at mild temperatures without generating hazardous subproducts and show an interesting selectivity.^15,16^ These enzymes are also capable of selectively oxidizing heteroatoms like sulfur, nitrogen, and boron.^17^ BVMOs require stoichiometric amounts of the expensive cofactors NADPH, and to a lesser extent NADH, for biotransformations. BVMOs can be classified into type I BVMOs, which are single-component enzymes using NADPH as a hydride donor and FAD as a prosthetic group, type II BVMOs, which use NADH and FMN and require two components (a reductase and an oxidase).^18−20^ Type I FMOs are single-component enzymes that utilize NADPH and FAD. Type II FMOs are also single-component enzymes and use NAD(*P*)H and FAD.^21,22^ Among the diverse groups of flavin-containing monooxygenases, type II flavin-containing monooxygenases have been receiving increasing attention as they display cofactor flexibility, accepting both NADH and NADPH.^23−25^ This feature makes the type II FMO subclass appealing for whole-cell biocatalysis as they can use both cofactors available in the cell.

To date, characterized type II FMOs have included SMFMO from *Stenotrophomonas maltophilia,*(26) PSFMO from *Pseudomonas stutzeri* NF13, and CFMO from *Cellvibrio* sp. BR.^27^ These three enzymes were described to catalyze mostly sulfoxidations and the Baeyer–Villiger monooxygenation of the model substrate bicyclo[3.2.0]hept-2-en-6-one (**1a**), with no conversion of other ketones such as cyclopentanone, cyclohexanone, acetophenone, and octan-2-one.^26^ Recently, BVMO-catalyzed sulfoxidation reactions were studied in detail by Bordewick et al.^21^ Crystal structures of the aforementioned enzymes are available, showing that they are homodimers. Other characterized type II FMOs are FMO-E, -F, and -G from *Rhodococcus jostii* RHA1.^17,28^ These enzymes also display cofactor promiscuity and have an N-terminal extension of approximately 160 residues when compared to SMFMO, CFMO, and PSFMO (Figure S1). This N-terminal extension has been proposed to facilitate BVMO activity in these enzymes, even though the mechanism for this remains unclear.^28^ FMO-E, -F, and -G perform Baeyer–Villiger monooxygenations in cyclobutanone-derived compounds and also catalyze monooxygenations of bicyclic ketones such as **1a** and norcamphor (**2a**), among others, but have not shown activity for cyclohexanone-derived ketones.^28^ A structural model of FMO-E was used to compare type I BVMOs (NADPH dependent) and type I FMO, uncovering that these classes use different strategies to incorporate oxygen into their substrates. While type I BVMOs have an arginine in the active site that is crucial for catalysis, FMO-E has no homologous active residue identified in that position. The authors describe a histidine and an arginine that possibly participate in the catalytic process, but mutagenesis experiments were inconclusive because the mutated enzyme was unable to retain the FAD. Finally, the type II FMOs PsFMO A, B, and C from *Pimelobacter sp*. Bb-B were described.^29^ These enzymes were active toward several cyclic ketones, such as some derived from cyclobutanone, cyclohexanone, 3-methyl cyclohexanone, camphor, and **1a**. PsFMOs also have an N-terminal extension similar to FMO-E, -F, and -G and display cofactor promiscuity.

To the best of our knowledge, no cold-active flavin-dependent monooxygenase has been described to date. Also, no type II FMOs similar to the ones mentioned above have been crystallized so far. Musumeci et al. identified putative Baeyer–Villiger monooxygenase (BVMO) sequences by metagenomic data mining from polar and subpolar marine sediments.^8^ In this study, modeling and docking procedures were used to structurally characterize these enzymes, identifying several promising features. Among them, the enzymes displayed a broader entrance to the active site, structural flexibility, and larger catalytic pockets compared to characterized BVMOs. These results, while only *in silico*, indicate that cold-active BVMOs are present in nature.

One of the main challenges for industrial utilization of BVMOs is the difficulty of supplying sufficient oxygen for the reaction. In fact, whole-cell biotransformations using BVMOs have been described as oxygen-limited at high cell concentrations (>2 g_dcw_ L^–1^).^30,31^ Fueled by the high oxygen solubility at lower temperatures, we sought to identify a cold-active BVMO that would permit facilitated oxygen supply. In this study, we report the first crystal structure and biochemical characterization of a type II FMO from the Antarctic bacterium *Janthinobacterium svalbardensis.* JsFMO shows unusual structural features among FMOs, including an N-terminal domain with an unclear role. The enzyme retains most of its activity at low temperatures and catalyzes the monooxygenation of sulfide compounds as well as linear and cyclic ketones with cofactor promiscuity, enabling low-temperature applications of BVMOs. We evaluated the performance of JsFMO in whole-cell biocatalysis utilizing the heterotrophic bacterium *E. coli*, which allows cofactor regeneration during the reaction.^32,33^ To the best of our knowledge, this is the first structural characterization of a cold-active enzyme capable of catalyzing Baeyer–Villiger monooxygenations.

## 2 Results and Discussion

### 2.1 Genome Mining in Microorganisms Isolated from the Antarctic

**Figure 1:**
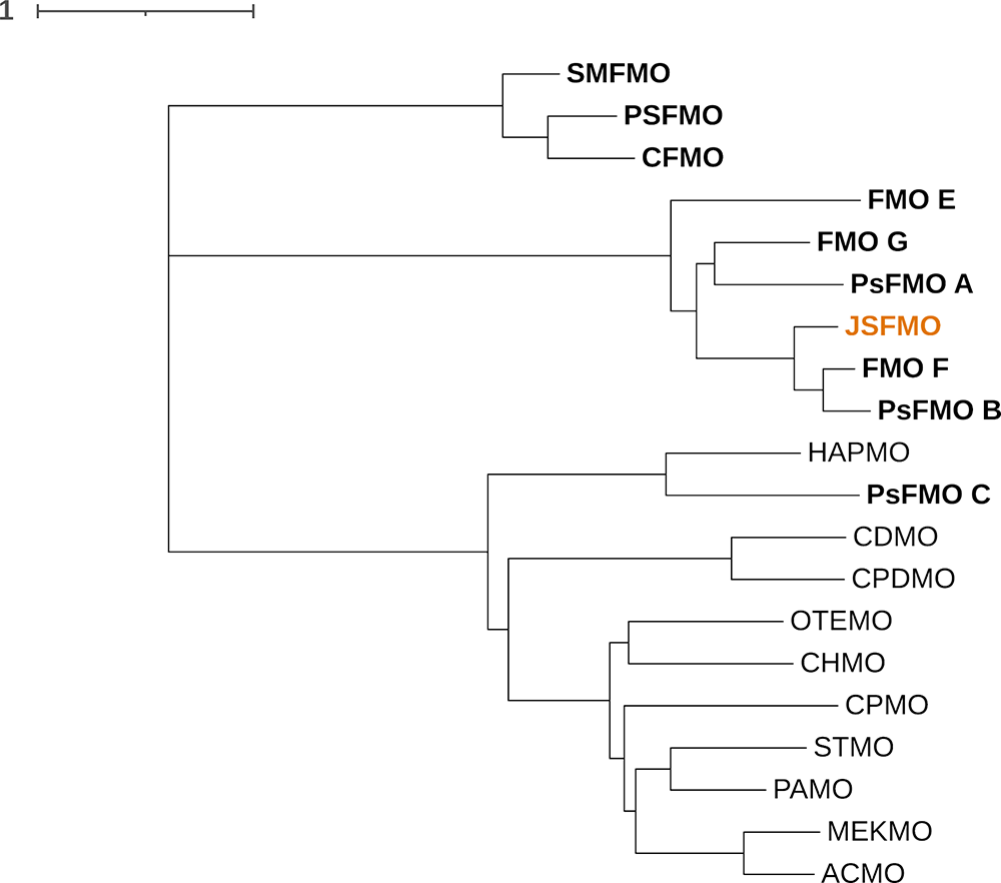
Phylogram showing type II FMO characterized to date (bold) and BVMO-I. JsFMO is shown in orange. The tree was constructed using Mega X^37^ and visualized with iTOL.^38^ The amino acid sequences of the enzymes shown are found in Table S1.

In order to find a cold-active flavin-dependent monooxygenase catalyzing Baeyer–Villiger monooxygenations, we selected genomes of organisms isolated from the Antarctic in the Integrated Microbial Genomes and Microbiomes database.^34,35^ When this study was conceived, no information about PsFMO A, B, and C was available; instead, we utilized FMO-E, FMO-F, and FMO-G from *Rhodococcus jostii* RHA1 as templates for a BLASTP search to find genes of putative cold-active BVMOs displaying cofactor promiscuity. A sequence from *Janthinobacterium svalbardensis* PAMC27463, isolated from a sweet water lake on King George Island in Antarctica, attracted our attention. The putative enzyme, JsFMO, shared the N-terminal extension present in FMO-E, -F, and -G and showed a percentage of identity of 36%, 71%, and 48% with these enzymes, respectively. JsFMO also displays the typical Rossmann fold and type I FMO motif (FxGxxxHxxx[YF][KR]) also found in other type II FMOs (Figure S1). We performed a phylogenetic analysis using amino acid sequences of BVMOs that showed that the enzymes with the N-terminal extension clustering together, except for PsFMO C, which appears to be more related to type I BVMOs such as 4-hydroxyacetophenone monooxygenase from *Pseudomonas fluorescens* ACB (HAPMO)^36^ (Figure 1). In contrast to other type I BVMOs, HAPMO also has an N-terminal extension, which has been described to play an important role in protein structural integrity.^36^ The shorter type II FMOs (SMFMO, PSFMO, and CFMO) cluster together in a separate clade. This clustering pattern relates well to the differences in substrate acceptance between type II FMOs with and without the N-terminal extension, as the last group shows higher activity toward sulfoxidation reactions and accepts a smaller number of ketones as substrates.

### 2.2 Recombinant Production and Stability Study of JsFMO

JsFMO was recombinantly produced and purified with a yield of ∼20 mg L^–1^ of culture, similar to the 25 mg L^–1^ reported for FMO-E.^28^ According to results obtained *via* size exclusion chromatography, JsFMO is a dimer (Figure S2). While the oligomeric state of type II FMOs with the N-terminal extension has not been described, SMFMO, CFMO, and PSFMO have their dimeric conformation in common with JsFMO.^17^

**Figure 2:**
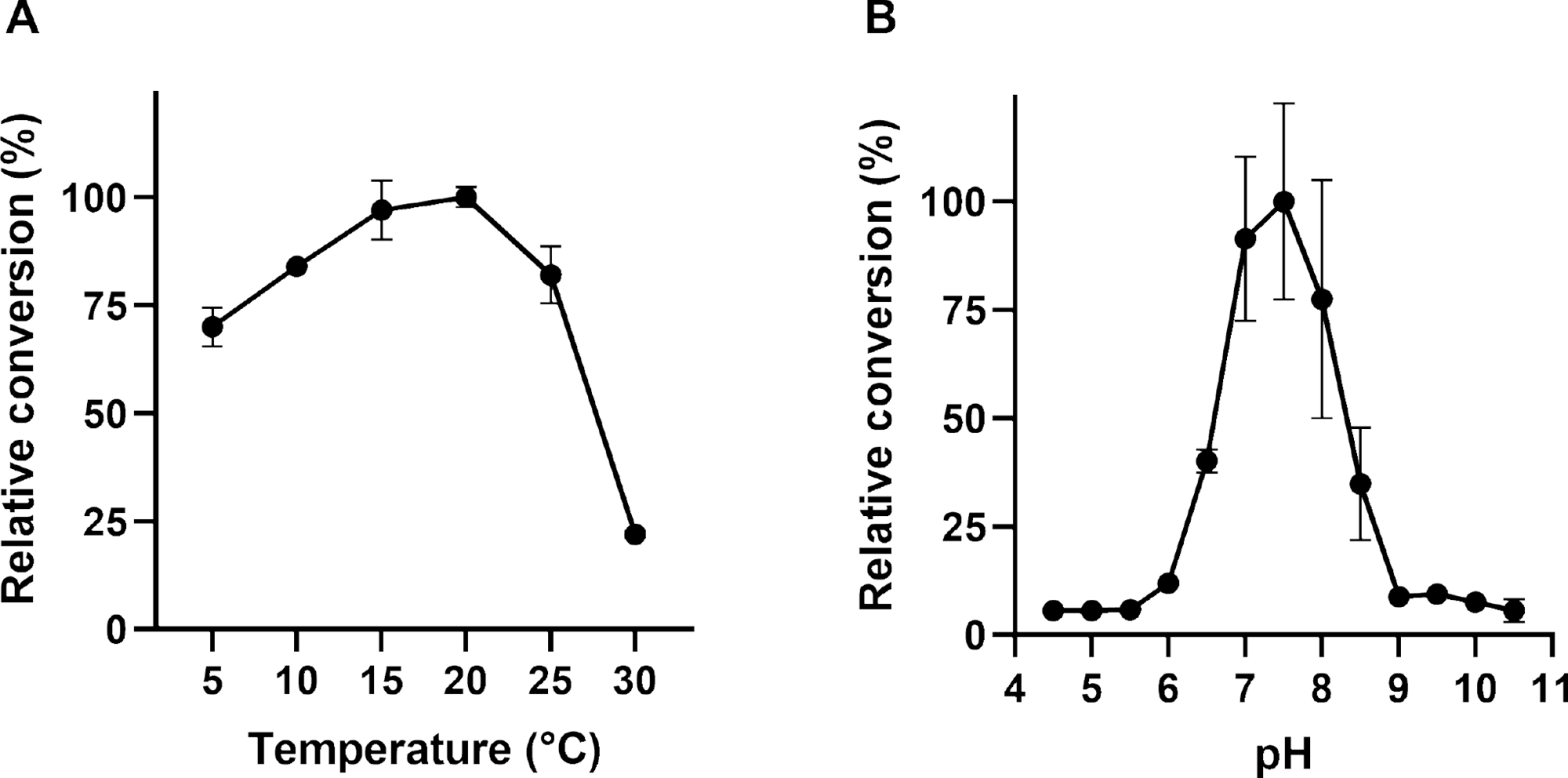
Relative conversion of JsFMO for **1a** after 4 h of reaction. (A) Values at different temperatures between 5 and 30 °C. (B) Values at different pH, ranging from 4.5 to 10.5.

To test the utility of JsFMO for biotransformation, reactions of **1a** together with a phosphite dehydrogenase (PTDH) for NADPH regeneration were performed for 4 h at different temperatures and pH values. The resulting activity profiles depend on the stability and specific activities of JsFMO and the regeneration system under the given conditions. The optimal temperature of this system was found at 20 °C, with the enzyme maintaining most of its activity in the range of 5 to 25 °C (Figure 2A). At higher temperatures, the relative conversion quickly dropped, with 82% at 25 °C and just 22% at 30 °C. The high retention of activity at very low temperatures (70% at 5 °C) and the quick loss of activity at temperatures above room temperature confirm our initial assumption that JsFMO is a cold-active enzyme. Unfortunately, no data is available for thermostability-related parameters of other type II FMOs to allow for a head-to-head comparison. In previous studies, reactions for PsFMO A, B, and C were carried out at 25 °C or 30 °C;^29^ in the case of FMO-E, F, and G, a temperature of 24 °C was used,^28^ and for SMFMO, PSFMO and CFMO, the enzymes were characterized at room temperature.^26,27^ We obtained a melting temperature (*T*_M_) of 34 °C for JsFMO using the ThermoFAD method.^39^ As a reference, the widely studied cyclohexanone monooxygenase (CHMO) from *Acinetobacter calcoaceticus* NCIMB 9871 has a *T*_M_ of 37 °C.^22^ This enzyme is well known for its broad substrate scope, but its use is hampered by its low stability. In fact, CHMO has a half-life of 72 h at 4 °C^22^ and 1 day at 25 °C.^40^ In contrast, JsFMO showed a significantly higher half-life at low temperatures, with a value of 20 days at 10 °C (Figure S3). The half-life time of JsFMO was also determined at 20 °C and was found to be 20.3 h (Figure S3). After incubation for 1 h at different temperatures and measuring residual activity, we obtained a *T*_50_^60^ of 29.2 °C (Figure S4). The activity of JsFMO is not high, and it is likely that enzyme engineering will be required to achieve competitive catalytic performance. Nevertheless, there is currently no rationale known for increasing enzyme activity at low temperatures. Therefore we believe that JsFMO represents an excellent basis for further engineering studies.^41,42^

The active site of cold-active enzymes is particularly flexible compared to the rest of the structure. Consequently, they are inactivated by heat even before they are unfolded, as they undergo a localized loss of structure in the area where the catalytic reaction occurs.^43^ This is not the case for mesophilic and thermophilic enzymes, where the activity of the enzyme is generally lost when the unfolding temperature is reached. Therefore, the *T*_50_^60^ has different implications for cold-active enzymes compared to their mesophilic and thermophilic counterparts. The optimal pH was determined to be 7.5, with some activity maintained between 6.5 and 8.5. (Figure 2B). This is within the range usually observed for type I BVMOs.

We evaluated the NADPH consumption rate of JsFMO in the absence of substrate (Table 1). The futile NADPH oxidase activity was 30 to 80 times lower than in the presence of the fast-converted substrate **1a**. The uncoupling reaction was comparable across the tested temperature range, while the initial substrate-dependent reaction rates showed a clear temperature dependence with the highest activities obtained at 25 °C.

**Table 1:** Specific Activities (U mg^–1^) of JsFMO at Different Temperatures in the Presence and Absence of **1a**^a^

temperature (°C)	with **1a**	without **1a**	ratio (+**1a**/–**1a**)
5 °C	0.246 ± 0.050	0.003 ± 0.001	82
10 °C	0.508 ± 0.020	0.017 ± 0.003	30
15 °C	0.568 ± 0.014	0.013 ± 0.009	44
20 °C	0.634 ± 0.011	0.019 ± 0.001	33
25 °C	0.799 ± 0.016	0.021 ± 0.003	38

a Reaction rates were determined by following the NADPH depletion at 340 nm.

**Figure 3:**
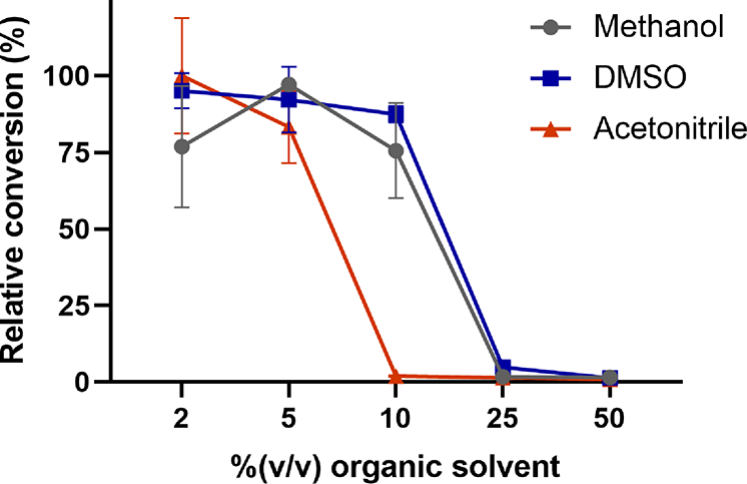
Conversion after 4 h of reaction using different concentrations of organic solvents for JsFMO with 2.5 mM of **1a**.

We also studied the effect of organic solvent exposure on JsFMO by carrying out the monooxygenation of **1a** for 2 h at different solvent concentrations (Figure 3). From these data, *C*_50_ values were calculated, defined as the concentration of solvent in buffer at which half-inactivation of the enzyme is observed. Acetonitrile was the least tolerated solvent, with a *C*_50_ of just 7% (v/v). The enzyme displays a higher tolerance toward DMSO and methanol, with a *C*_50_ of 17% (v/v) and 15% (v/v), respectively. Secundo et al. determined *C*_50_ for CHMO and the thermostable phenylacetone monooxygenase (PAMO) from *Thermobifida fusca* while exposed to different organic solvents;^44^ they obtained *C*_50_ for methanol of 55% and 7% with PAMO and CHMO, respectively. For acetonitrile, *C*_50_ was 22% for PAMO and 6% for CHMO. However, these results cannot be directly compared as Secundo et al. measured the specific activity following the change in absorbance at 340 nm for a relatively short period of time while we carried out the reaction for 4 h. A longer period of exposure to the organic solvent probably has a higher impact on the retained activity of the enzyme. Notwithstanding the differences in the methods to obtain the values, the *C*_50_ of 7% obtained with acetonitrile indicates that JsFMO has a slightly higher tolerance for acetonitrile and methanol in comparison to CHMO but may not be substantially more stable than other BVMOs in organic solvents.

### 2.3 Crystal Structure of JsFMO

**Figure 4:**
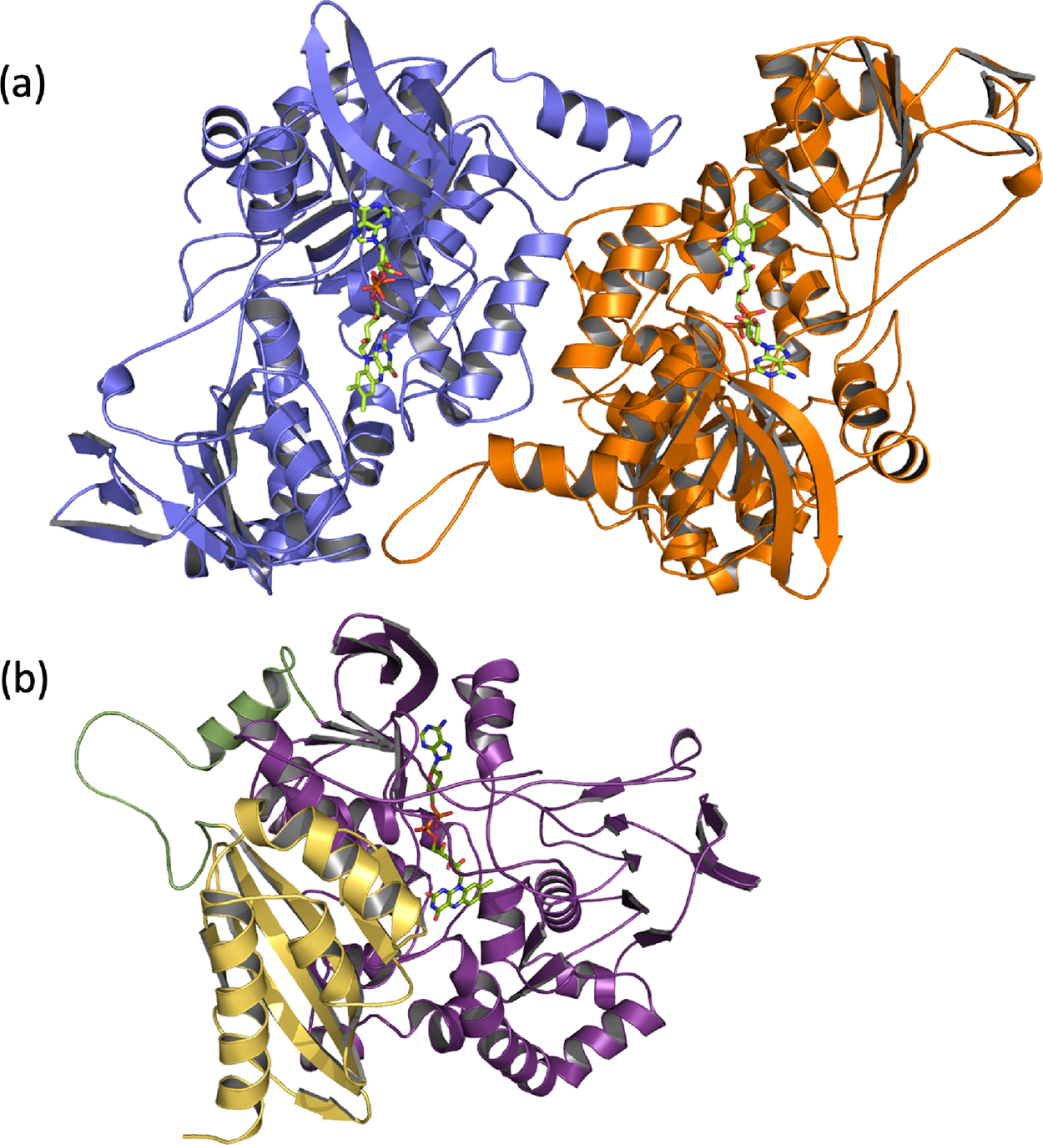
(a) Dimer of JsFMO. (b) Monomer of JsFMO divided by domains: N-terminal (yellow), linker loop (green), and monooxygenase domain (purple). The structure can be found under the PDB code: 8ACS.

In the crystal structure, JsFMO appears as a homodimer (Figure 4a), with an extended dimerization interface of approximately 2300 Å^2^, consistent with results from size exclusion chromatography (Figure S2). Four protomers forming two dimers are present in the asymmetric unit. Each protomer appears to be divided into two domains (Figure 4b); an N-terminal domain with a SnoaL-like fold^45^ and a C-terminal monooxygenase domain (MoD), bearing two typical Rossman folds for FAD and NAD(*P*)H binding. The two domains are in contact with each other and are connected by a long loop (residues 126–150), a short alpha helix (residues 151–161), and a short unstructured “spacer” (residues 162–170). Chains A and D show electron density for all protein residues, excluding the C-terminal His-tag and the first 5 and 6 residues at the N-terminus, respectively. Chains B and C miss electron density for some residues of the long loop connecting the N-terminal domain to the monooxygenase domain. While chains A, B, and C are highly similar with respect to the protein and the cofactor, chain D shows some differences. First, the isoalloxazine ring and part of the ribityl chain of the FAD appear rotated by approximately 15° (Figure S5); furthermore, the isoalloxazine ring appears to slide slightly forward, reducing the space between the flavin and the side-chain of His-216. Another notable difference is the movement of the NADPH-binding domain (Figure S5) that, in chain D, seems to tilt toward the active site. This transition looks very similar to the one observed in CHMO for the “open” and “closed” states, which probably resemble different stages of the catalytic cycle.^46^ No NADP^+^ is present in the crystal structure, so the movement of this domain must be due to its intrinsic flexibility.

#### 2.3.1 N-Terminal Domain

**Figure 5:**
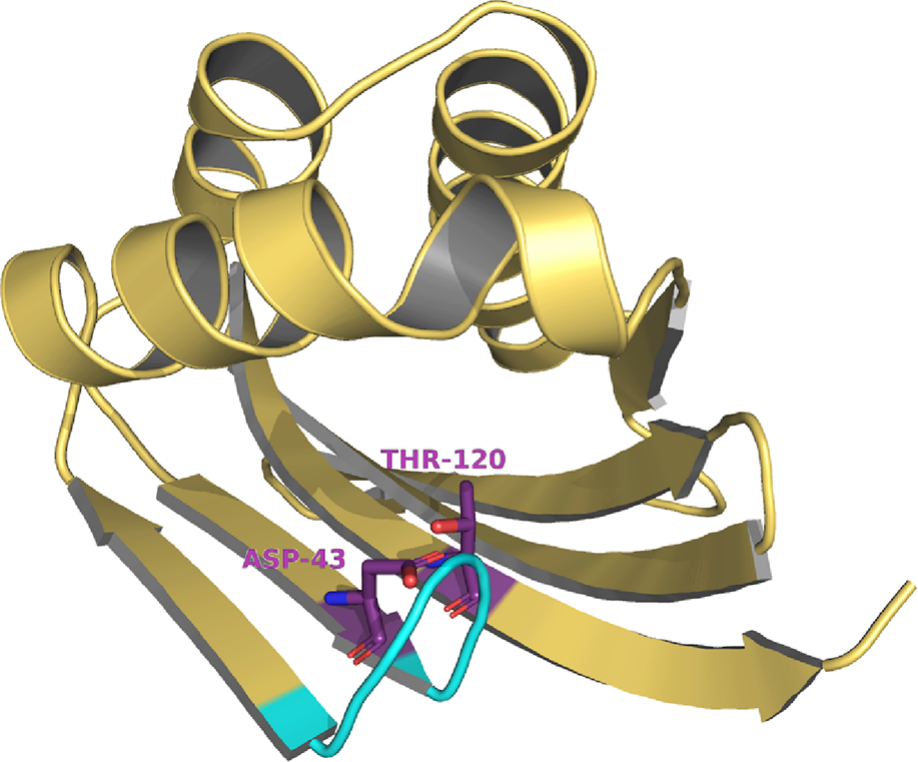
N-terminal domain of JsFMO. The loop blocking the entrance to the cavity is represented in cyan.

The N-terminal domain is the most characteristic and puzzling part of this protein. The domain appears to have a SnoaL-like fold,^45^ as predicted by the Pfam server and the homology model built with AlphaFold.^47^ A structural comparison using the DALI server^48^ shows indeed similarities with proteins bearing this fold (e.g., ketosteroid isomerases, NTF2-like superfamily proteins, and limonene-1,2-epoxide hydrolase). Similar to ketosteroid isomerases, the N-terminal domain presents a highly hydrophobic core, with the only polar residues being Asp-43 and Thr-120 (Figure 5). Asp-43 occupies the same position as Asp-38 in ketosteroid isomerase, a residue known to be critical for catalysis in this enzyme.^49,50^ Despite these similarities, Thr-120 does not occupy a position that would suggest a catalytic mechanism similar to the one of ketosteroid isomerases; furthermore, the access to these residues is prevented by the loop formed by the residues 44–51 (Figure 5). We note that the N-terminal domain does not present extensive contacts with the MoD; also, the long loop connecting the two domains allows an extensive degree of freedom to the N-terminal domain, which might move away from the MoD core. This leaves the possibility that the loop 44–51 is folding back in the putative active site just in the crystal structure, thus not excluding a possible catalytical activity. Contrary to what was initially proposed,^28^ the N-terminal domain seems unlikely to be involved in the Baeyer–Villiger monooxygenase activity since it is quite distant from the active site of the MoD.

Part of the loop is also involved in the dimerization, and in this region, the protein establishes several interchain contacts (Arg-136-Glu-385, Arg-148-Glu-353, and several H-bonds between backbone atoms).

#### 2.3.2 Monooxygenase Domain

To highlight similarities with other known structures, we also performed a structural comparison with the DALI server on the monooxygenase domain. The software found structural similarities with PSFMO,^27^ SMFMO,^26^ bacillithiol disulfide reductase (Bdr) from *Staphylococcus aureus*,^51^ AncFMO2, and AncFMO5.^52^ Apart from the last two, most of the similarities are due to the two Rossmann folds involved in FAD and NAD(*P*)H binding, which appear completely superimposable to the ones of the above-mentioned proteins, as well as the ones from CHMO^53^ and PAMO.^54^ In the NADPH binding domain, the typical consensus sequence (GxGxxG) is replaced by GxNxxA, as previously reported also for FMO-F from *R. jostii,* an enzyme with which JsFMO has in common its cofactor promiscuity and preference toward NADPH.^17^ The NAD(*P*)H-binding domain bears the arginine residue typically involved in the binding of 2′-phosphate (Figure S6). Another common structural feature is a C-terminal helix (567–586), although this one has no sequence similarity with the other enzymes. Apart from that, the structure of the MoD of JsFMO appears to be different from the known class B BVMO and type II FMO structures.

#### 2.3.3 The Active Site

**Figure 6:**
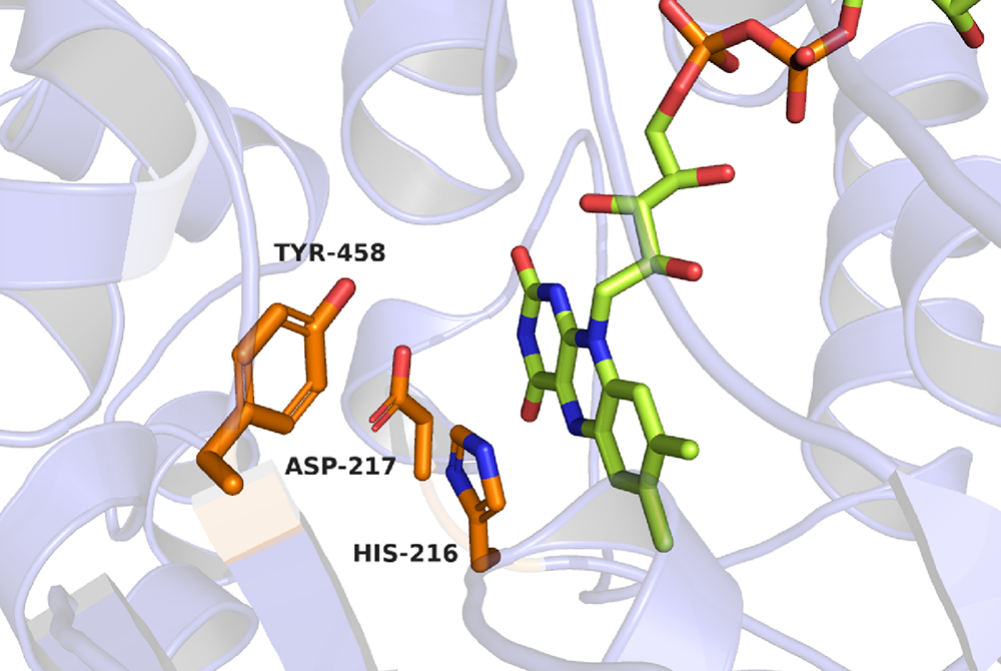
Active site of JsFMO.

The active site of JsFMO does not resemble those of known monooxygenases. In type I BVMOs, an arginine residue is known to be critical for catalysis. Fraaije et al. proposed that Arg-563 might be involved in the reaction mechanism of FMO-E.^28^ This arginine is conserved in JsFMO (Arg-571) and has a critical structural role, coordinating residues from different secondary structure elements of the protein (Figure S7). It is plausible that replacing this residue with alanine in FMO-E did lead to the loss of the cofactor and activity.^28^ Apart from that, no other arginine residue is present in the active site of JsFMO (Figure 6). Instead, Tyr-458 occupies a similar position to the catalytic arginine of type I BVMOs and Glu-281 in the ancestral flavin-containing monooxygenase 2. It must be noted that the side chain of the BVMO’s arginine points toward the flavin and it is close to the C4a atom, while Tyr-458 occupies a more distant position, comparable to Glu-281 in AncFMO2. Mattevi et al. showed that replacing this residue with histidine did not affect the sulfoxidation but did also not introduce any BVMO activity; on the other side, AncFMO5 H282E completely lost the ability to perform Baeyer–Villiger monooxygenations.^52^ As suggested by them, a hydrogen bond donor in this position is likely critical for BVMO activity, but several adaptations are required for the formation of the Criegee intermediate. The presence of a hydrogen-bond donor (Tyr-458 in JsFMO) suggests a reason why the enzyme can perform both reactions. Furthermore, this tyrosine is conserved in all the type II FMOs with the N-terminal extension investigated so far, indicating its importance.

**Figure 7:**
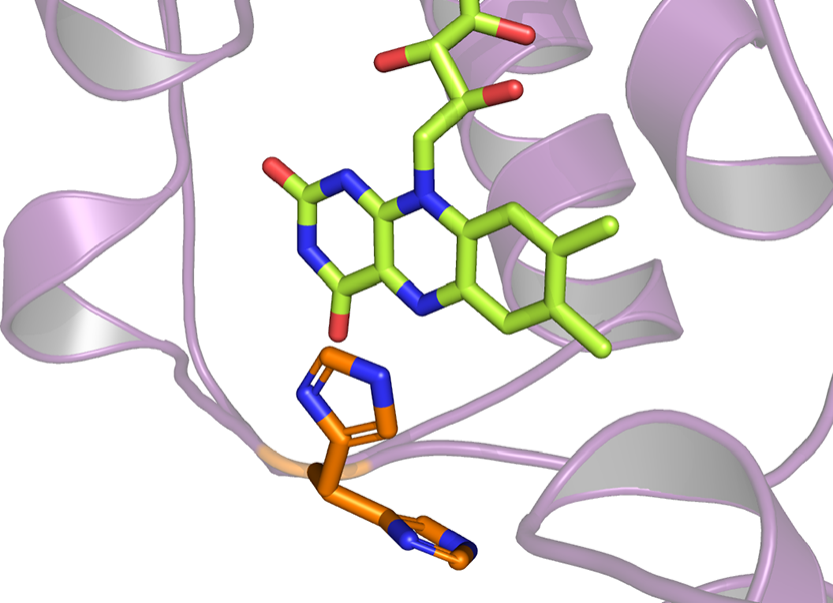
Alternate conformations of His-216 in chain D.

Closer to the reactive center of the enzyme, we find His-216 and Asp-217, which both might be involved in the reaction mechanism. Asp-217 sits on the side of the isoalloxazine ring and establishes an H-bond with the N3-atom of the flavin. An aspartate residue in a similar position can also be found in CHMO (Asp-59) and PAMO (Asp-66); nonetheless, in these enzymes, the aspartate occupies a position much closer to the C4a. In JsFMO, the aspartate is positioned farther away from C4a and might only have a marginal role in catalysis. His-216 instead occupies a quite interesting position. Differently from any previously known flavoprotein monooxygenase, this residue is located right in front of the N5-atom of FAD. The distance between the N5 of the flavin and His-216 ranges from 3.5 Å (Chain A) to 2.9 Å (Chain D). In the observed conformation, this residue would thus likely prevent a hydride transfer from NADPH to the FAD. In chain D, however, this residue is also rotated in an alternate conformation (Figure 7). This rotation would clear the space for the entrance of NADPH, thus allowing the reduction of the cofactor and the formation of the peroxyflavin. Fraaije et al. reported the loss of the FAD when they replaced this residue with alanine in FMO-E.^28^ Nonetheless, replacing H216 with alanine in JsFMO did not lead to any FAD loss (see next paragraph), thus allowing us to characterize the effect on the activity of this mutation.

##### 2.3.3.1 Amino Acid Variants of the Active Site

To test the role of the residues mentioned above, we generated four variants: H216N, H216A, D217A, and Y458F. The FAD loading estimated from the UV–vis absorbance spectra was comparable for both JsFMO wild-type and variants (78–82%); furthermore, no significant changes were observed in the UV–Vis spectra of the variants (Figure S8). The consumption of NADPH in the absence of the substrate was very low for all the variants and comparable to the one of the wild type (Table S2). The effect on the Baeyer–Villiger oxidation of **1a** indicates the involvement of some of these residues in the catalytic activity. The substitution of H216 with an asparagine slightly reduced enzyme activity (0.705 ± 0.012 U mg^–1^ versus 0.799 ± 0.016 U mg^–1^ of the wild type), suggesting that the acid–base chemistry of the histidine is not critical for the catalysis. Contrary to this, the H216A variant had an almost two-fold drop in activity (0.457 ± 0.015 U mg^–1^). Although this is not a drastic change, it indicates that a hydrogen-bond donor in this position is important, probably for the correct positioning of the nicotinamide moiety of the NAD(*P*)H. A similar two-fold drop in activity was measured for the D217A (0.444 ± 0.043 U mg^–1^) and Y458F (0.418 ± 0.042 U mg^–1^). While none of the proposed residues is essential for the activity, H216, D217, and Y458 appear to be involved in the catalysis. As mentioned in the previous paragraph, it is likely that several adaptations are required for the formation of the Criegee intermediate; thus, the effect of a single amino acid exchange might not be drastic.

##### 2.3.3.2 A Tunnel for the Substrate

**Figure 8:**
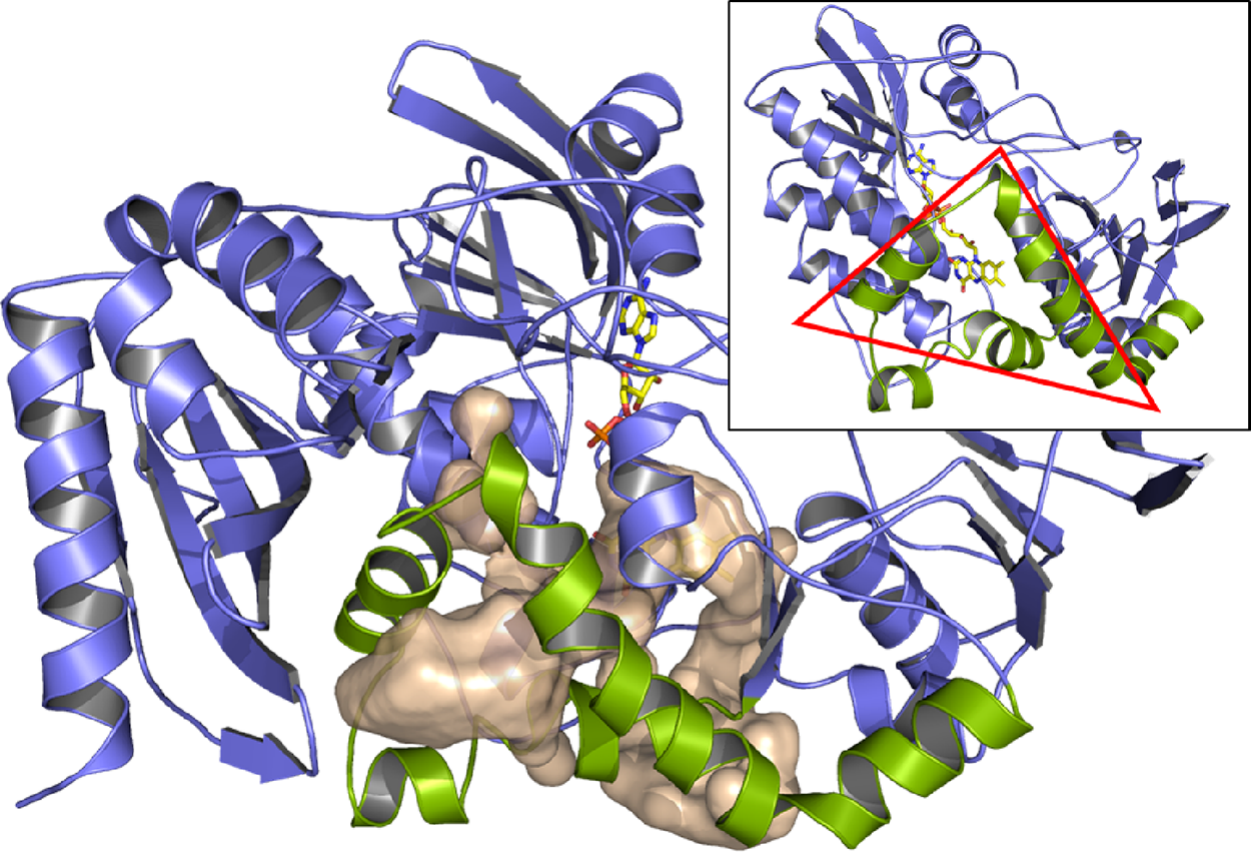
Tunnel leading to the active site, calculated using the HOLLOW script. The entrance is highlighted in green.

In the observed structure, the active site appears completely inaccessible, except for the NADPH binding site, where a cavity grants the NAD(*P*)H to reach the flavin. In BVMOs and NMOs (recently inserted in the same phylogenetic group of type II FMOs^55^), the NAD(P)^+^ occupies this pocket for the complete catalytic cycle;^56,57^ therefore, it is very likely that the substrate enters the active site on a different way. A triangular-shaped helical structure (residues 370–434) appears to create an opening to the active site (Figure 8). Curiously, a similar structure has been recently reported by Mattevi et al. in their ancestral reconstruction of mammalian monooxygenases.^52^ Although the sequence similarity in these regions is low, we decided to explore the presence of a tunnel using the program HOLLOW.^58^ The calculation indicates the presence of a tunnel that begins from the abovementioned helical structure and leads to the active site in front of the flavin. The pocket extends toward the bottom of the active site behind the flavin and terminates at the dimerization interface in our representation. In the dimer AC, the access to the surface is obstructed by the loop 125–151 of the other protomer, but in the dimer BD, the loop of chain B is unfolded, and the tunnel is accessible (Figure S9). The rotation of His-216 blocks access to the final part of the tunnel (Figure S9b). In the absence of additional data, we thus speculate that the substrate must enter the active site from the triangle-shaped helical structure and probably leaves the cavity in the same way after the monooxygenation.

#### 2.3.4 Structural Rationalization of Cold Activity

Enzymes evolve cold activity through different strategies, but higher structural flexibility is generally believed to be the main factor.^59,60^ It is difficult to rationalize the cold activity of JsFMO based on its structure alone. Therefore, we generated AlphaFold models of three type II FMOs from the mesophilic bacterium *Rhodococcus jostii* RHA1^61,62^ (FMO-E, FMO-F, and FMO-G) and a type II FMO from the thermophilic organism *Actinomadura rubrobrunea.*(63) The comparison of these models with the crystal structure of JsFMO did not show an increase in unstructured regions (as judged by the proportions of loop regions and ordered secondary structure elements). In addition, the amino acid sequence of JsFMO does not contain a higher proportion of glycine, alanine, or threonine residues than the other proteins.

Other features, still related to structural flexibility, include a decrease in the number of hydrogen bonds and salt bridges.^64,65^ We analyzed these parameters in the five enzyme structures using the program Hbplus v3.2.^66^ The number of hydrogen bonds (per 100 residues) was not significantly different between JsFMO and three of the four mesophilic or thermophilic enzymes (Figure S10A). Curiously, the mesophilic FMO-G from *R. jostii* showed the lowest H-bond content. On the other hand, the JsFMO structure contained a significantly lower number of salt bridges than the other proteins (Figure S10B). Overall, the results of the comparisons do not provide a definitive, structural explanation of the cold activity of JsFMO. However, the reduced number of salt bridges may at least serve as an indicator.

### 2.4 Substrate Scope, Selectivity, Cofactor Usage and Kinetics

**Scheme 1:**
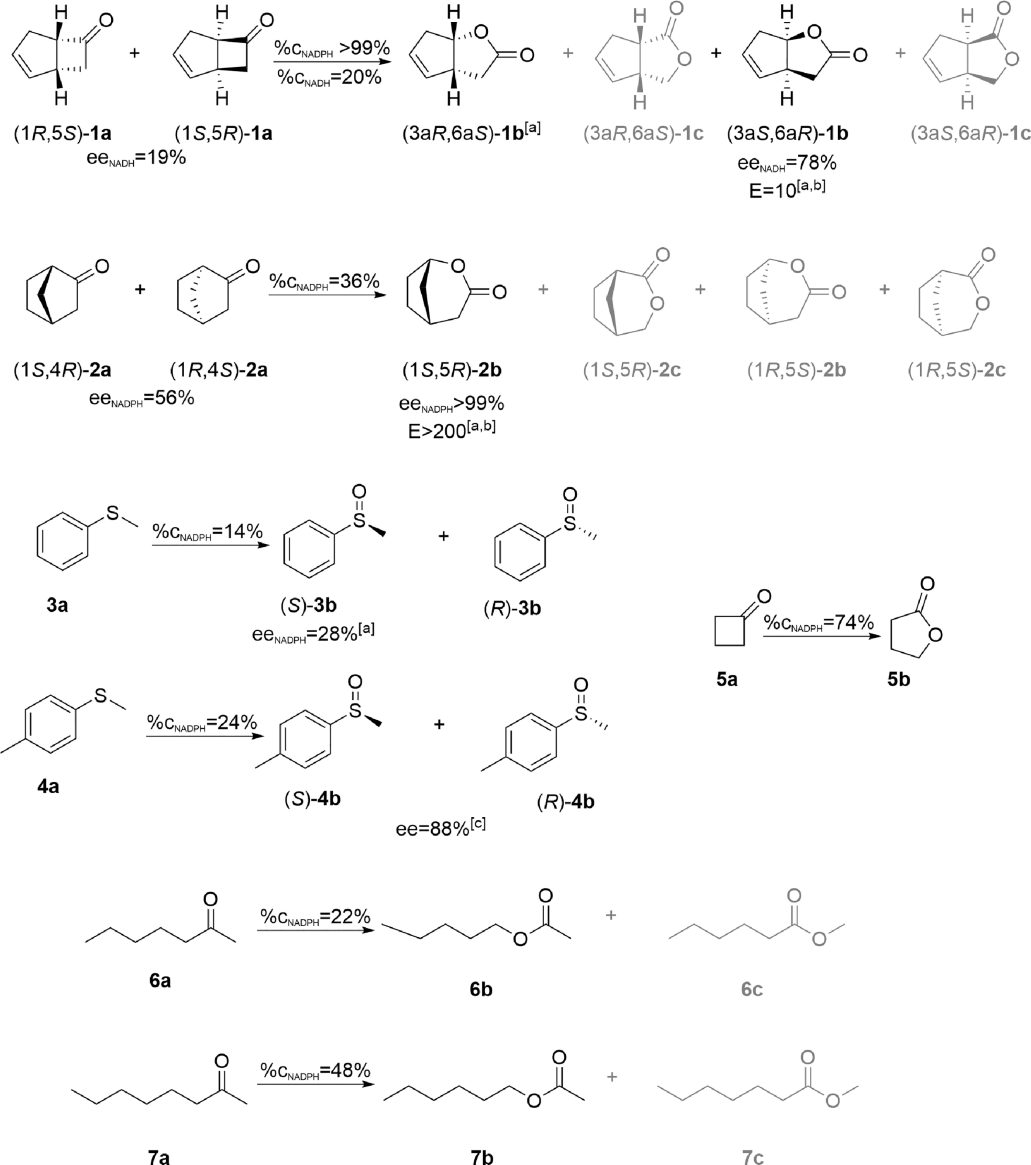
Substrates accepted by JsFMO and their corresponding products Potential products of the reaction that were not detected are shown in gray. (^[a]^The absolute configuration of the enantiomers of **1a**, **1b**, **2a**, **2b**, and **3b** was identified by catalyzing the oxidation with CHMO and comparing the elution order in chiral gas chromatography to literature.;^68−70^^[b]^*E* value calculated according to Straathop and Jogejan.;^71^^[c]^First eluting peak according to gas chromatography analysis.)

We evaluated the substrate scope of JsFMO using a set of sulfides and linear and cyclic ketones and detected product formation using gas chromatography–mass spectrometry analyses (GC–MS) (Tables S3 and S4). Substrates accepted by JsFMO and their corresponding products are shown in Scheme 1. The best-accepted substrate for JsFMO was the bicyclic **1a**, with a conversion of >99% and 20% after 24 h of reaction at 10 °C using NADPH or NADH as a cofactor, respectively. A comparison of kinetic constants showed that JsFMO prefers NADPH (*k*_cat_/K_M_ = 14.6 mM^–1^ s^–1^) over NADH (*k*_cat_/K_M_ = 2.9 mM^–1^ s^–1^) (Figure S11). While the turnover numbers with both cofactors are comparable (*k*_cat(NADPH)_ = 0.21 ± 0.1 s^–1^; *k*_cat(NADH)_ = 0.23 ± 0.1 s^–1^), the affinity of JsFMO for NADPH (K_M_ = 14.4 ± 2.5 μM) was much lower than for NADH (K_M_ = 78.5 ± 7.5 μM). As mentioned above, JsFMO bears the arginine residue typically involved in recognizing the 2′-phosphate of NADPH (Arg-363);^36,67^ enzymes preferring NADH usually show non-charged residues in the same position, like glutamine or threonine.^26^ It is, in any case, hard to define the cofactor preference just based on single amino acid variations. As pointed out by Bornscheuer et al.,^67^ the preference toward NADH or NADPH is defined by the hydrogen-bonding network of the whole pocket (including water molecules); mutating residues not in direct contact with the NAD(*P*)H might favor a cofactor over the other.

Chiral GC analysis revealed that the enzyme prefers the enantiomer (1*S*,5*R*)-**1a** as the substrate and displays high regioselectivity, with only the normal lactones (**1b**) being detected after 24 h (Scheme 1, Table 1). The enzyme also accepts substrate **5a**, with 74% conversion after 24 h. The good activity with **1a** and **5a** indicates that JsFMO converts cyclobutanone derivates, which was also stated for other type II FMOs with an N-terminal extension. Bulkier monocyclic ketones were also tested, but no conversion was detected for cyclopentanone, cyclohexanone, cyclooctenone, and some other derived compounds (Table S2). It is curious that the enzyme accepted norcamphor (**2a**) as the substrate but did not accept the related compounds camphor (with additional methyl groups on C1,7,7) or fenchone (C1,3,3), possibly indicating the interference of these methyl groups with the proper positioning of the substrate in the active site. JsFMO displayed high enantioselectivity and regioselectivity with **2a**, with only one of the enantiomers of the normal product (1*S*,5*R*) being detected (ee_S_ = 56%, ee_P_ = 99%, *E* > 200) at 53% conversion. JsFMO also catalyzed the monooxygenation of some medium-length linear ketones (**6a** and **7a**). In the case of these last two substrates, only the acetate products were identified (**6b** and **7b**), indicating that JsFMO is highly regioselective for these two compounds. Interestingly, while other type II FMOs with an N-terminal extension showed some conversion with phenylacetone,^28^ no conversion was detected with JsFMO using the closely related compounds propiophenone, valerophenone, 4-methylpropiophenone, or 1-phenyl-2-butanone. In the case of the sulfides, the enzyme displayed poor conversions, with 14% for **3a** and 24% for **4a** after 24 h of reaction and an enantiomeric excess of the product ee_p_ of 28% and 88% respectively, with a preference for the (*S*) enantiomer in the case of **3a** (Scheme 1, Table 2). While the conversions obtained for most of the assayed substrates are low in comparison to other BVMOs, the substrate scope and stability of JsFMO make this enzyme a potentially good scaffold for activity improvement or substrate scope widening for cold-active applications.

**Table 2:** Specific Activities for Whole-Cell Biotransformations with 7 Different Substrates in *E. coli* at 10 and 25 °C

	specific activity (mU mg_dcw_^–1^)	
substrate	*E. coli* 10 °C	*E. coli* 25 °C
**1a**	5.8 ± 0.4	9.3 ± 1.7
**2a**	4.4 ± 0.4	1.4 ± 0.8
**3a**	0.079 ± 0.004	0.095 ± 0.016
**4a**	0.059 ± 0.007	0.065 ± 0.005
**5a**	1.2 ± 0.1	1.8 ± 0.4
**6a**	0.08 ± 0.01	0.21 ± 0.2
**7a**	0.17 ± 0.03	0.38 ± 0.1

Kinetic parameters were determined for **1a** spectrophotometrically at room temperature. Under these conditions, JsFMO has a *k*_cat_ of 0.29 ± 0.01 s^–1^ and K_M_ 1.05 ± 0.23 mM (Figure S12). Comparatively, PsFMO A has a K_M_ of 1.51 mM for **1a**,^29^ similar to the one obtained for JsFMO, while the K_M_ of FMO-E, with a value of 19.8 mM, is considerably higher.^52^ FMO-E compensates for this issue with a higher *k*_cat_ of 2.7 s^–1^, displaying a kinetic efficiency of 136 M^–1^ s^–1^, while the catalytic efficiency is 812 M^–1^ s^–1^ for PsFMO A and 276 M^–1^ s^–1^ for JsFMO, respectively. Unexpectedly, JsFMO has the lowest K_M_ of the reported type II FMOs, as cold-active enzymes tend to have higher K_M_ values than their mesophilic counterparts.^43^

### 2.5 Whole-Cell Biocatalysis

**Figure 9:**
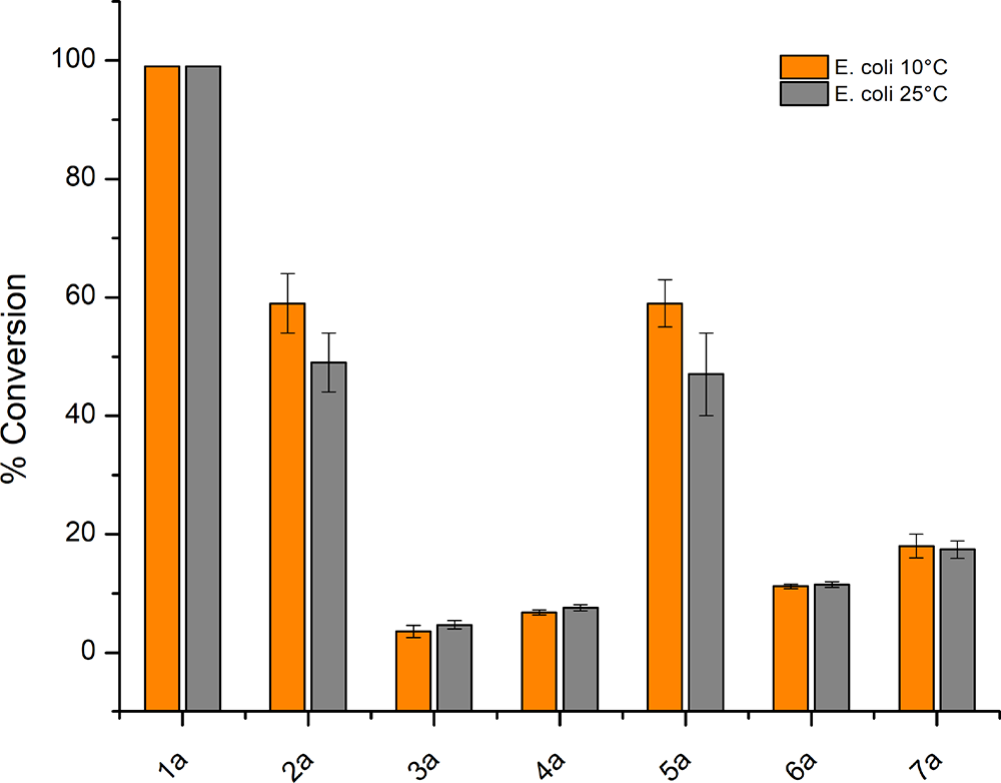
Conversions after 24 h for whole-cell biotransformations in *E. coli* with 7 different substrates at 10 and 25 °C.

After the *in vitro* characterization of JsFMO and to overcome the issue of adding stoichiometric amounts of NAD(*P*)H during Baeyer–Villiger monooxygenations, we studied whole-cell biocatalysis in recombinant *E. coli* as a heterotrophic model for NAD(*P*)H regeneration. The conversions and specific activities of the whole-cell biocatalysts toward seven substrates were determined at 10 °C, to study the feasibility of low-temperature applications, and at 25 °C, considering situations when avoiding additional heating might be desired (Figure 9, Table 2). **1a** was the tested substrate that performed best, with the full conversion being achieved after 24 h for both *E. coli* conditions tested, followed by **2a** and **5a**, which were converted faster at 10 °C than at 25 °C. Conversions for **3a**, **4a**, **6a**, and **7a** were all below 20%; nevertheless, the conversions observed at 10 °C with *E. coli* show that whole-cell Baeyer–Villiger monooxygenations at low temperatures and in conditions that permit higher oxygen solubility are, in principle, possible.

In order to test other systems for cofactor regeneration, we also tried biotransformations in the cyanobacterium *Synechocystis sp*. PCC6803. Cofactor availability of NADP(H) is reported to be more abundant than NAD(H) in cyanobacteria while the NAD(H) pool is usually higher in heterotrophic organisms.^72^ However, the intolerance of *Synechocyatis sp*. PCC6803 to cold temperatures and the toxicity of the studied compounds^73^ led to poor results in conversion, and yields were lower than in whole-cell biotransformations in *E. coli* (Figure S13).

## 3 Conclusions

In this study, we reported the structure and the biochemical characterization of the first cold-active type II FMO with flexible cofactor acceptance. JsFMO displays good activity and stability that enables its use in the low range of temperatures. The enzyme accepts sulfides and linear and cyclic ketones as substrates and catalyzes the oxidation of the substrates with outstanding regioselectivity. The high enantioselectivity toward **2a** demonstrates that the generally higher flexibility of the active site of cold-active enzymes does not necessarily reduce the selectivity of these enzymes. The enzyme shows the classic folds found in FMOs together with new structural features, as the N-terminal domain, with its role still an open question. The monooxygenase domain curiously resembles the active site of the monooxygenases from higher eukaryotes, with Tyr-458 and His-216 as catalytic residues and a triangular-shaped helix creating the entrance to the active site. This is an example of convergent evolution, as the structure seems to have independently evolved in superior eukaryotes and bacteria. Whole-cell biotransformations at 10 and 25 °C in *E. coli* showed that whole-cell biocatalysis for cofactor regeneration of JsFMO is possible both at room temperature and at colder temperatures where the solubility of oxygen is higher. BVMO reactions at a technical scale using cell-free extracts have been reported previously,^14^ and we also expect that reaction and enzyme engineering will be promising strategies to improve the activity and selectivity of JsFMO.

## 4 Experimental Section

### 4.1 Materials

All chemicals were bought from Sigma-Aldrich (Austria), except for bicyclo[3.2.0]hept-2-en-6-one (Alfa Aesar, Germany).

### 4.2 Phylogenetic Tree

The multiple sequence alignment and phylogenetic tree were done using MegaX.^37^ The multiple sequence alignment was done by ClustalX with default settings and visualized using Snapgene 5.1.5. The evolutionary history was inferred by using the Maximum Likelihood method and Le_Gascuel_2008 model.^74^ Initial trees for the heuristic search were obtained automatically by applying Neighbor-Join and BioNJ algorithms to a matrix of pairwise distances estimated using the JTT model and then selecting the topology with superior log likelihood value. A discrete Gamma distribution was used to model evolutionary rate differences among sites (5 categories (+G, parameter = 2.5283)). The rate variation model allowed for some sites to be evolutionarily invariable ([+I], 1.09% sites).

### 4.3 Protein Expression and Purification

The JsFMO gene was ordered in pET-28a(+)-TEV vector, codon-optimized for expression in *E. coli* from GenScript Biotech (Netherlands). Enzyme variants were generated by site-directed mutagenesis using the QuikChange kit (Thermo Fisher Scientific). All constructs were used to transform *E. coli* ArcticExpress (DE3) chemo-competent cells (Agilent Technologies, USA), which is a genetically optimized host for enzyme production at low temperatures.

For expression, 1 L of TB media with 40 mg L^–1^ kanamycin was inoculated with 25 mL of preculture. The culture was incubated at 37 °C, 130 rpm for 3.5 h (OD_600_ between 0.6 and 0.8). 1 mM isopropyl β-d-1-thiogalactopyranoside (IPTG) was used to induce the expression. According to the manual, the flasks were incubated at 12 °C, 130 rpm for 24 h. After that, cells were harvested by centrifuging 15 min at 6370*g*, 4 °C (Beckman Coulter, USA, JA10 rotor) and resuspended in 20 mM Tris–HCl, 500 mM NaCl, 10% glycerol, 1 mM DTT, 50 μM FAD, 20 mM imidazole, pH 7.5. Cells were sonicated for 6 min, output control 7, duty cycle 70% and then centrifuged at 27220*g* for 1 h to get the cell-free extract.

For purification, an ÄKTA pure system (GE Healthcare Life Sciences, Austria) operated at 4–8 °C was used. The obtained supernatant was loaded on a pre-equilibrated His-Trap FF crude 5 mL column (GE-Healthcare, Austria). The loaded column was washed with 20 mM Tris–HCl, 500 mM NaCl, 10% glycerol, 50 μM FAD, and 20 mM imidazole, and then the purified enzyme was eluted using the same buffer with 300 mM imidazole concentration. Fractions containing purified JsFMO were dialyzed overnight with buffer 100 mM Tris–HCl, 500 mM NaCl, 10% glycerol, 1 mM DTT, pH 7.5 and stored at −80 °C.

For protein crystallization, an additional size-exclusion chromatography step was carried out. The protein was loaded on a HiLoad 16/60 Superdex 200 prep grade column mounted on an ÄKTA-Pure system (GE Healthcare Life Sciences, Austria). The elution was performed at a flow rate of 0.5 mL min^–1^ using 20 mM TRIS buffer, pH 7.5, containing 100 mM NaCl and 20 μm FAD. The eluted fractions were concentrated for further use in crystallization. Protein purification and purity of enzymes were checked by SDS-PAGE analysis for all variants (Figures S14–S18).

### 4.4 Protein Crystallization

For screening and optimization, the sitting-drop vapor diffusion method was used. The protein solution was concentrated to 8.6 mg/mL and screened against Morpheus I (Molecular Dimensions), Index (Hampton Research), and JCSG+ (Molecular Dimensions) screens on a 96-well 3 Lens crystallization plate (SwissCI). The plates were incubated in the cold room (4–8 °C) and checked regularly. Needle-shaped crystals were obtained in conditions D12 and E11 after 5 days. The screen was optimized to obtain the best condition: 16 mg/mL JsFMO mixed 2:1 with the reservoir (glycerol 20%, PEG 8000 13%, 100 mM Bis-Tris pH 6.5). After optimization, the quality of the crystals was further improved using Crystallophore Lu-X04 (Polyvalan, France).^75^ All the screens had to be performed manually in the cold room due to the protein instability at room temperature.

### 4.5 Data Collection and Structure Solution

Crystals were harvested after 10 days and flash-frozen in liquid nitrogen. Due to the high glycerol concentration in the crystallization solution, no additional cryoprotectant was applied. Diffraction data were collected at the DESY beamline P11 at a wavelength of 1.34 Å. The indexing of the collected dataset was done with the CCP4 cloud service.^76^ The structure was solved by molecular replacement (MR) using Phenix Phaser,^77^ with the best-ranked relaxed model built with AlphaFold^48^ as a search template. The MR solution was refined using Phenix refine.^77^ After the first refinement, FAD was added to the molecules in Coot.^78^ After several rounds of refinement, *R*_work_ and *R*_free_ values could not be improved, so the refinement was considered concluded. The final *R*_work_ and *R*_free_ values were 18.2% and 22.9%, respectively. Details about the refinement and data statistics can be found in the Supplementary Material (Table S5).

### 4.6 Optimal pH and Temperature Determination

The optimal pH was determined using 200 μL of cell-free extract prepared in 10 mM Tris–HCl, 500 mM NaCl, 10% glycerol, 1 mM DTT, pH 7.5. The reaction mix contained 5 mM bicyclo[3.2.0]hept-2-en-6-one, 0.1 mM NADPH, 3 μM PTDH, 10 μM FAD, and 10 mM Na_2_HPO_3_·5H_2_O. The reaction was carried out at 1 mL total volume using buffer 500 mM NaCl, 10% glycerol, 1 mM DTT and 100 mM citrate buffer (pH 4.5, 5 and 5.5), potassium phosphate buffer (pH 6, 6.5 and 7) or Tris–HCl (pH 7.5, 8, 8.5 and 9) at 10 °C and 600 rpm, in triplicate. 200 μL of sample was taken at 0 and 4 h for GC analysis.

The optimal temperature was determined in a similar manner using 2.5 mM **1a**, 5 mM NADPH, 2.5 μM FAD, and 2.5 μM purified JsFMO. Reactions were carried out in triplicate at 600 rpm and temperatures between 5 and 30 °C and extracted after 4 h for GC analysis.

### 4.7 Organic Solvent Tolerance

The reactions contained the following: 250 μL of cell-free extract, 5 mM NADPH, 2.5 mM **1a**, and buffer 100 mM Tris–HCl, 500 mM NaCl, 10% glycerol, 10 μM FAD, 1 mM DTT, pH 7.5 to complete 500 μL. Different substrate stocks were prepared in the analyzed organic solvents to have the desired solvent percentage when adding 2.5 mM **1a** to the mix. After 4 h of reaction, 200 μL of the sample was extracted for GC analysis. The reactions were carried out at 10 °C and 600 rpm in triplicate.

### 4.8 Melting Temperature, *T*_50_^60^, and Half-life Time Determination

For determination of the melting temperature, the ThermoFAD method was used.^39^ Briefly, purified JsFMO was diluted to a concentration of approximately 30 μM. 25 μL of the sample was heated from 4 to 100 °C using a real-time PCR system (BioRad, Austria). The melting temperature was obtained at the minimum of the derivative of the signal profile.

For *T*_50_^60^ determination, purified JsFMO was incubated at 5, 10, 15, 20, 22.5, 25, 27.5, 30, and 32.5 °C for 1 h using a thermal cycler. After that, 5 μM of the enzyme was added to a reaction mix containing 10 μM PTDH, 100 μM NADPH, 10 mM Na_2_HPO_3_·5H_2_O, 2 mM **1a**, 100 mM Tris–HCl, 500 mM NaCl, 10% glycerol, 1 mM DTT, 5 μM FAD at pH 7.5 and incubated at 10 °C, 600 rpm for 2 h. 200 μL of the sample was taken for GC analysis. For half-life time determination, the procedure was the same as for *T*_50_^60^ but the enzyme was incubated at 10 or 20 °C and samples were taken at different time points.

### 4.9 Kinetic Parameters

Kinetic parameters were determined by following the NADPH or NADH depletion in an Eon plate reader (Biotek, Germany) at 340 nm (ε = 6220 M^–1^ cm^–1^) and room temperature unless specified otherwise. The reaction mix contained 0.25 mM NADPH, 0.5–5 μM JsFMO, **1a** in concentrations ranging from 0.25 to 20 mM in 100 mM Tris–HCl buffer, pH 7.5, containing 500 mM NaCl, 10% glycerol, 1 mM DTT, and 5 μM FAD. The reaction was started by adding the enzyme, and absorbance at 340 nm was measured for 10 min. All concentrations of substrate were assayed in triplicate. The rates obtained from the linear slope of the curves were used for nonlinear fit to a Michaelis–Menten model with the OriginPro 2019b software (OriginLab, Northampton, MA, USA).

### 4.10 Cloning in SynRekB_cpc, Segregation in *Synechocystis sp*. PCC6803, and Whole Cell Biotransformations

The JsFMO gene was cloned in SynRekB plasmid with the cpc promotor^79^ using the restriction enzymes XhoI and NdeI (Thermo Scientific, Germany). pET-28a(+)-TEV-*jsfmo* and cpc::SynRekB plasmids were digested according to the manufacturer’s instructions. Digestion products were loaded into a 1% agarose gel for electrophoresis, and the corresponding bands were cut and purified from the gel. The fragments were subsequently ligated using T4 ligase (Thermo Scientific, Germany), and *E. coli* top 10 cells were transformed. Transformed cells were incubated in LB plates with 40 mg L^–1^ at 37 °C overnight. Colonies were sequenced to confirm the correct incorporation of the *jsfmo* gene.

For transformation and segregation of *Synechocystis*, wild-type cells were grown in BG-11 media at 140 rpm, room temperature, under white light (∼60 μE m^–2^ s^–1^) at 50% humidity till an OD_750_ of ∼1 was reached. 1.5 mL of cells was centrifuged at 2200*g* for 20 min, resuspended in 0.5 mL of fresh BG-11, and mixed with 5 μL of the construct. Cells were incubated for 4 h, 300 rpm, 30 °C in the dark and were then plated onto transfer membranes in BG-11 agar plates. The next day, the membrane was transferred to BG-11 plates with 25 mg L^–1^ kanamycin. During the next weeks, colonies were re-plated with increasing concentrations of kanamycin. After reaching 100 mg L^–1^ kanamycin, total segregation was confirmed by PCR.

For biotransformations in *Synechocystis*, 600 mL of BG-11 was inoculated with the JsFMO containing *Synechocystis* to an initial OD_750_ 0.05–0.1. Cells were incubated at room temperature and 130 rpm with a light intensity of approximately 100 μE m^–2^ s^–1^ under blue-red LED lamps. Cultures were harvested at an OD_750_ between 1 and 2 by centrifugation for 25 min at 3220*g* and resuspended with fresh BG-11 to a final OD_750_ of 10. Reactions were carried out in a total volume of 5 mL, using 5 mM of a substrate prepared in DMSO (final concentration: 0.5%). Reactions were carried out at approximately 150 μE m^–2^ s^–1^ using a blue-red lamp at 20 or 25 °C and 130 rpm in triplicate. 200 μL of the sample was taken at different time points for GC analysis.

### 4.11 Biotransformations in *E. coli* and with Purified Enzyme

For whole-cell biotransformations with *E. coli*, enzyme production was carried out as described in the protein expression and purification section. Cells were harvested by centrifuging for 20 min at 6370*g* and 4 °C and then resuspended in 50 mM Tris–HCl, 20 mM glucose, pH 8 to a final OD_600_ of 10. Reactions were carried out in a total volume of 5 mL, using 5 mM of a substrate prepared in DMSO (final concentration biotransformation: 0.5%), at 10 or 25 °C and 130 rpm. 250 μL of the sample was taken at different time points for GC analysis.

Reactions for determining conversions with the purified enzyme contained 5 μM JsFMO, 2 mM substrate, 10 μM PTDH, 10 mM Na_2_HPO_3_·5H_2_O, 0.1 mM NADPH in buffer 100 mM Tris–HCl, 500 mM NaCl, 10% glycerol, 1 mM DTT, 5 μM FAD, pH 7.5. Samples of 250 μL were taken at times 0 and 24 h and extracted for GC analysis.

### 4.12 Gas Chromatography-Flame Ionization Detector (GC-FID) Analysis

Samples were extracted in a ratio of 1:2 with dichloromethane containing 1 mM acetophenone as the internal standard. After discarding the aqueous phase, samples were dried with Na_2_SO_4_ and transferred to glass vials with 250 μL inlets. Details for the columns used, programs, and retention times are given in Table S4. For chiral identification of **1a**, **1b**, **2a**, **2b**, and **3b**, the Baeyer–Villiger monooxygenations were performed with CHMO, and the obtained results were compared to the literature.^68−70^

## Supporting Information Available

Multiple sequence alignment of type II FMOs; calibration curve for size exclusion chromatography; half-life time determination at 10 and 20 °C; depiction of residues involved in catalysis of JsFMO; absorption spectra of oxidized JsFMO and variant enzymes; kinetic constants for *Js*FMO with NADPH and NADH; kinetic parameters for JsFMO with **1a**; conversions and specific activities obtained in whole-cell biotransformations with *Synechocystis and E. coli;* SDS gels of the purification of JsFMO and variant enzymes (PDF)

## Abbreviations

BVMO: Baeyer–Villiger monooxygenase
FMO: flavin-containing monooxygenase
JsFMO: type II flavin-containing monooxygenase from *Janthinobacterium svalbardensis*
SMFMO: type II flavin-containing monooxygenase from *Stenotrophomonas maltophilia*
PSFMO: type II flavin-containing monooxygenase from *Pseudomonas stutzeri* NF13
CFMO: type II flavin-containing monooxygenase from *Cellvibrio* sp. BR.
FMO-E, -F, -G: type II flavin-containing monooxygenase from *Rhodococcus jostii* RHA1
PsFMO A, B, C: type II flavin-containing monooxygenase from *Pimelobacter sp*. Bb-B
CHMO: cyclohexanone monooxygenase from *Acinetobacter calcoaceticus* NCIMB 9871
PAMO: phenyl acetone monooxygenase from *Thermobifida fusca*
HAPMO: 4-hydroxyacetophenone monooxygenase from *Pseudomonas fluorescens* ACB
GC–MS: gas chromatography–mass spectrometry
GC-FID: gas chromatography-flame ionization detector
MoD: monooxygenase domain
MR: molecular replacement
