# Supplementary material for: A Cold-Active Flavin-Dependent Monooxygenase from Janthinobacterium svalbardensis Unlocks Applications of Baeyer–Villiger Monooxygenases at Low Temperature
Source: ACS Catal. 2023 Feb 27;13(6):3549–62. doi: 10.1021/acscatal.2c05160 (PMC10028610; doi:10.1021/acscatal.2c05160)
Supplement: Supplementary file 1 — cs2c05160_si_001.pdf [file cs2c05160_si_001.pdf]

Supplementary Information

A Cold-Active Flavin-Dependent Monooxygenase from *Janthinobacterium svalbardensis* Unlocks Applications of Baeyer-Villiger Monooxygenases at Low Temperature.

A.M. Chánique[a,b]#, N. Polidori[c]#, L. Sovic[a], D. Kracher[a], L. Assil-Companioni[a,d], P. Galuska[c], L.P. Parra[e], K. Gruber[c], R. Kourist*[a,c]

[a] Dr. A.M. Chánique, L. Sovic Msc., Dr. D. Kracher, L. Assil-Companioni, Prof. R. Kourist
Institute of Molecular Biotechnology
Graz University of Technology, NAWI Graz, BioTechMed-Graz
Petersgasse 14
8010 Graz (Austria)
E-mail: kourist@tugraz.at

[b] Dr. A.M. Chánique
Department of Chemical and Bioprocesses Engineering
School of Engineering
Pontificia Universidad Católica de Chile
Vicuña Mackenna 4860
7810000, Santiago (Chile)

[c] N. Polidori MSc, P. Galuska MSc., Prof. K. Gruber
Institute of Molecular Biosciences
University of Graz, NAWI Graz, BioTechMed Graz
Humboldtstraße 50
8010 Graz (Austria)
E-mail: karl.gruber@uni-graz.at

[d] Dr. L. Assil-Companioni, Prof. R. Kourist
ACIB GmbH
Petersgasse 14/1
8010, Graz (Austria)

[e] Prof. L. P. Parra
Institute for Biological and Medical Engineering
Schools of Engineering, Medicine and Biological Sciences
Pontificia Universidad Católica de Chile
Vicuña Mackenna 4860
7810000, Santiago (Chile)
E-mail: lparraa@ing.puc.cl

Contents

Figure S1. Multiple sequence alignment of characterized type II FMO 3

Figure S2. Calibration curve for size exclusion chromatography and table with the oligomeric state of JsFMO 4

Figure S3. Half-life time determination at 10°C (**A**) and 20°C (**B**) 4

Figure S5. The differences between chain A (yellow) and chain D (purple) 5

Figure S6. The arginine involved in 2’-phosphate binding of JsFMO 7

Figure S7. Arginine 571 and its network of contacts connecting different parts of the protein 7

Figure S8. Absorption spectra of oxidized JsFMO (A) and variant enzymes 8

Figure S9. The final part of the tunnel in the dimer AC (a) and BD (b) 9

Figure S10. Comparison of polar interactions in different type II FMOs 10

Figure S11. Kinetic constants for *Js*FMO with NADPH (a) and NADH (b) 11

Figure S12. Kinetic parameters for JsFMO with **1a**. 12

Figure S13. Conversions and specific activities obtained in whole cell biotransformations with *Synechocystis and E. coli*. 13

Figure S14. SDS gel of the purification of JsFMO. 14

Figure S15. SDS gel of the Immobilized Metal affinity-chromatography purification of JsFMO H216A 15

Figure S16. SDS gel of the Immobilized Metal affinity-chromatography purification of JsFMO H216N. 16

Figure S17. SDS gel of the Immobilized Metal affinity-chromatography purification of JsFMO Y458F 17

Figure S18. SDS gel of the Immobilized Metal affinity-chromatography purification of JsFMO D217A. 18

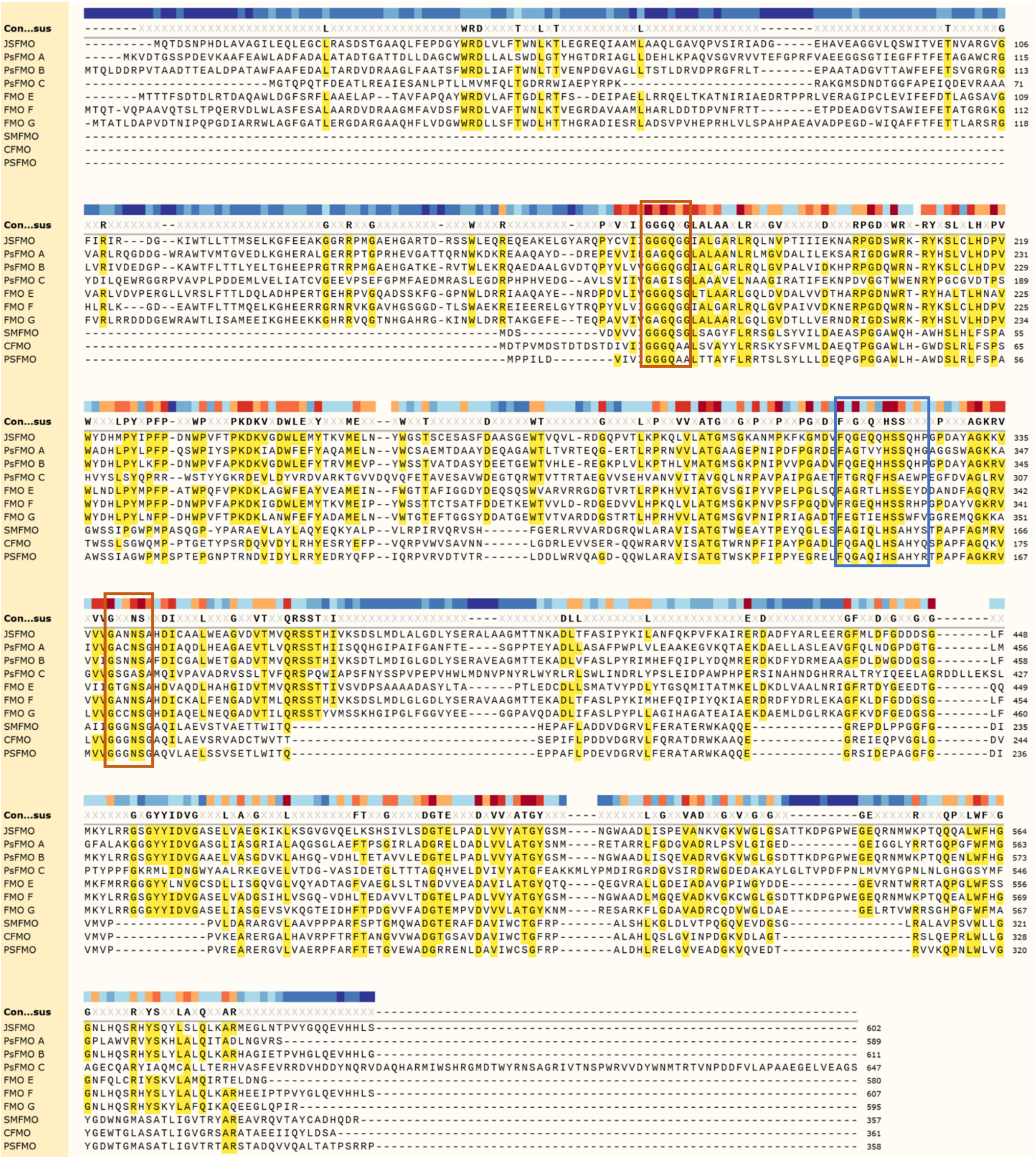

Figure S1. Multiple sequence alignment of characterized type II FMO. The alignment was done using Mega X and visualized with SnapGen 5.1.5. Consensus sequence is shown when more than 50% of the residues in a certain position matched. Residues matching the consensus are highlighted in yellow. Amino acids matching the consensus sequence are highlighted in yellow. The blue square shows the Type I FMO motif (FxGxxxHxxx[YF][KR]) and red squares indicate the two Rossmann fold motifs (GxGxxx(G/A)).

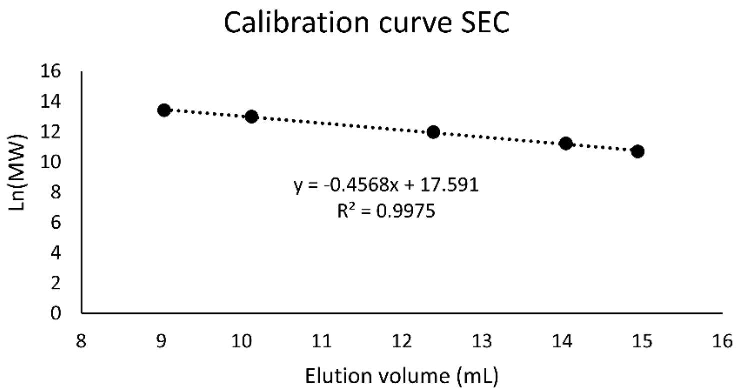

	Retention time (min)	Molecular weight according to calibration curve (Da)	Theorical molecular weight of the monomer (Da)	Oligomeric state
**JsFMO**	12.73	130333	68867	1.9

Figure S2. Calibration curve for size exclusion chromatography and table with the oligomeric state of JsFMO.

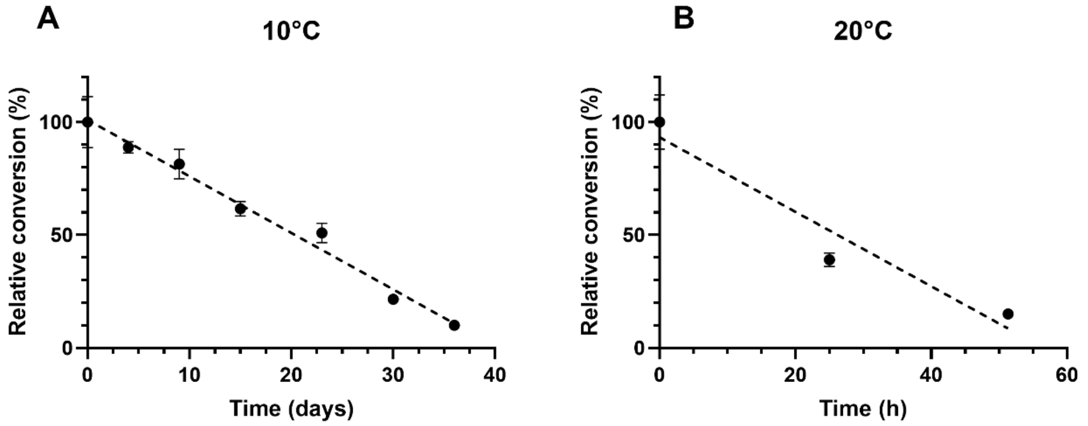

Figure S3. Half-life time determination at 10°C (**A**) and 20°C (**B**). Interpolated half-life time corresponds to 20 days for 10°C and 20.3 h for 20°C.

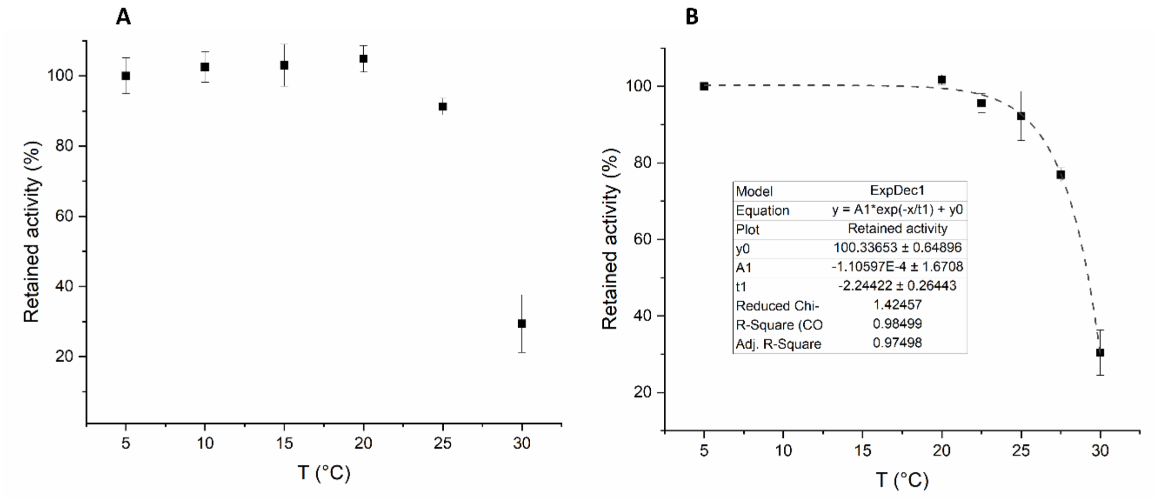

Figure S4. T_{50}^{60} determination. Residual activity was measured after 1h of incubation at the temperatures shown in the figure. The incubated enzyme was used for the monooxygenation of 2 mM of **1a** for 2h at 10°C. **A.** Measured every 5°C range 5° to 30°. **B.** Incubated every 2.5°C from 20 to 32.5°C. Protein evidently precipitated at temperatures above at 32.5°C, therefore, the reaction was not carried with this sample. Exponential decay model used for nonlinear fitting using OriginPro 2019b. According to the model, T_{50}^{60} = 29.2°C

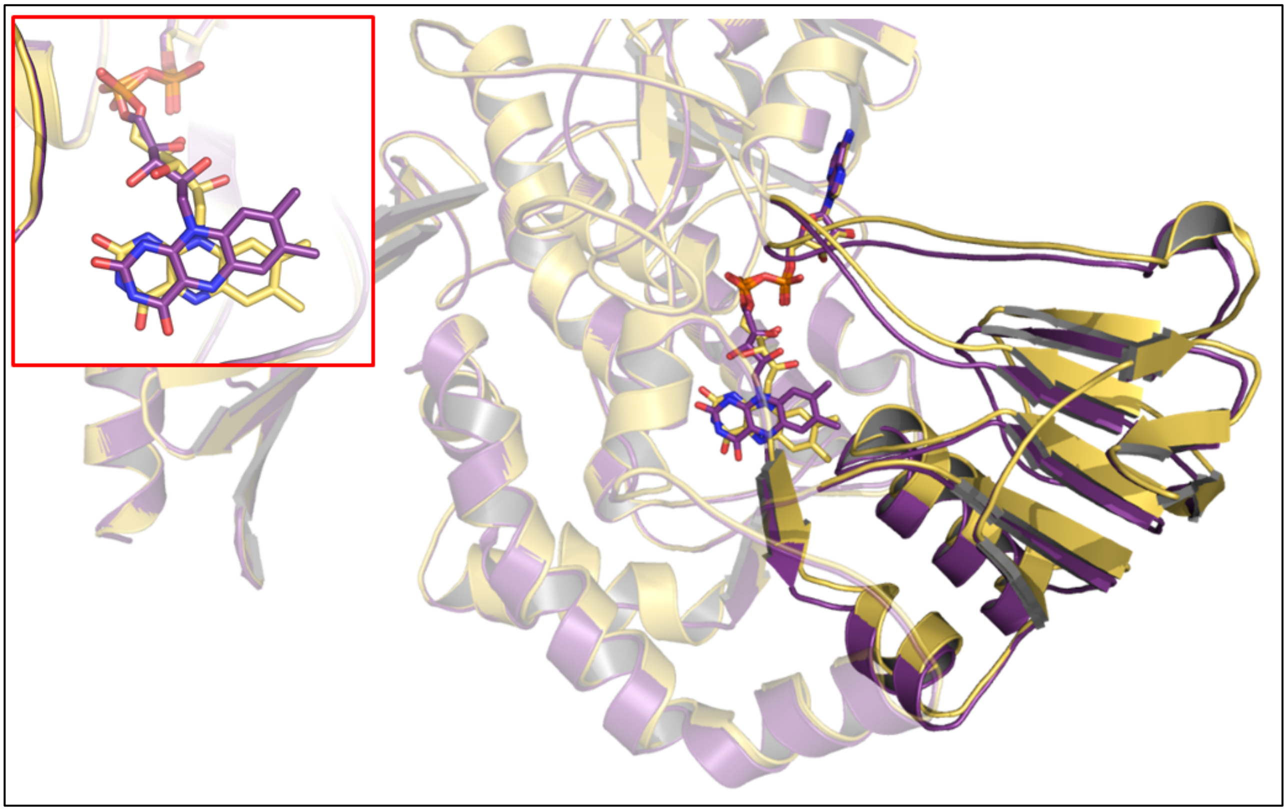

Figure S5. The differences between chain A (yellow) and chain D (purple).

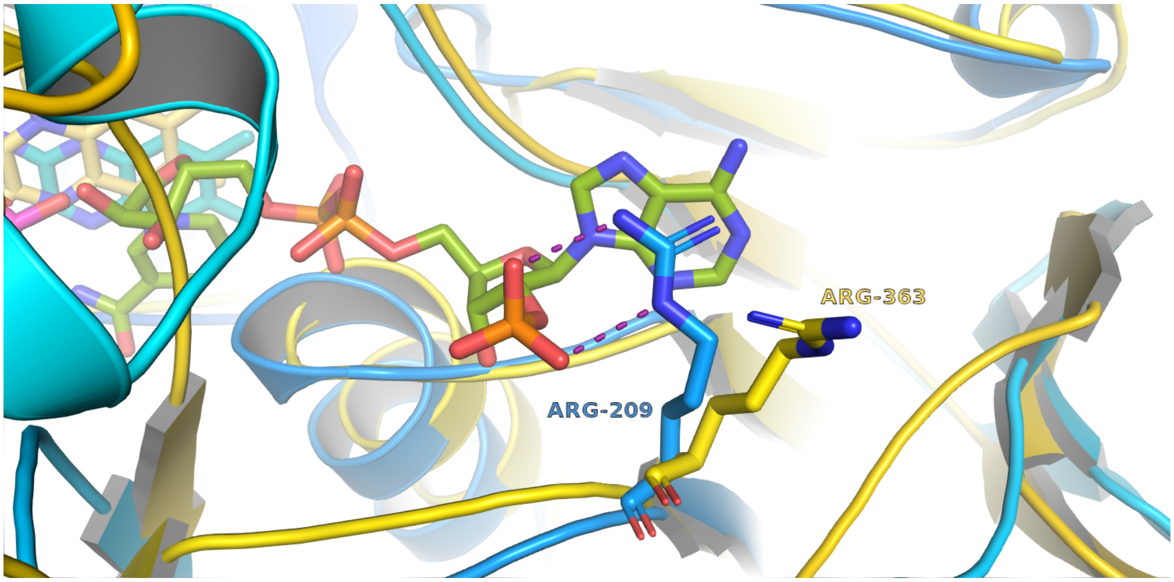

Figure S6. The arginine involved in 2'-phosphate binding of JsFMO (Arg-363, yellow) compared to the one of cyclohexanone monooxygenase from *Rhodococcus sp.* HI-31 (Arg-209, cyan). The structure of cyclohexanone monooxygenase is available on the PDB (4RG3)

Figure S7. Arginine 571 and its network of contacts connecting different parts of the protein.

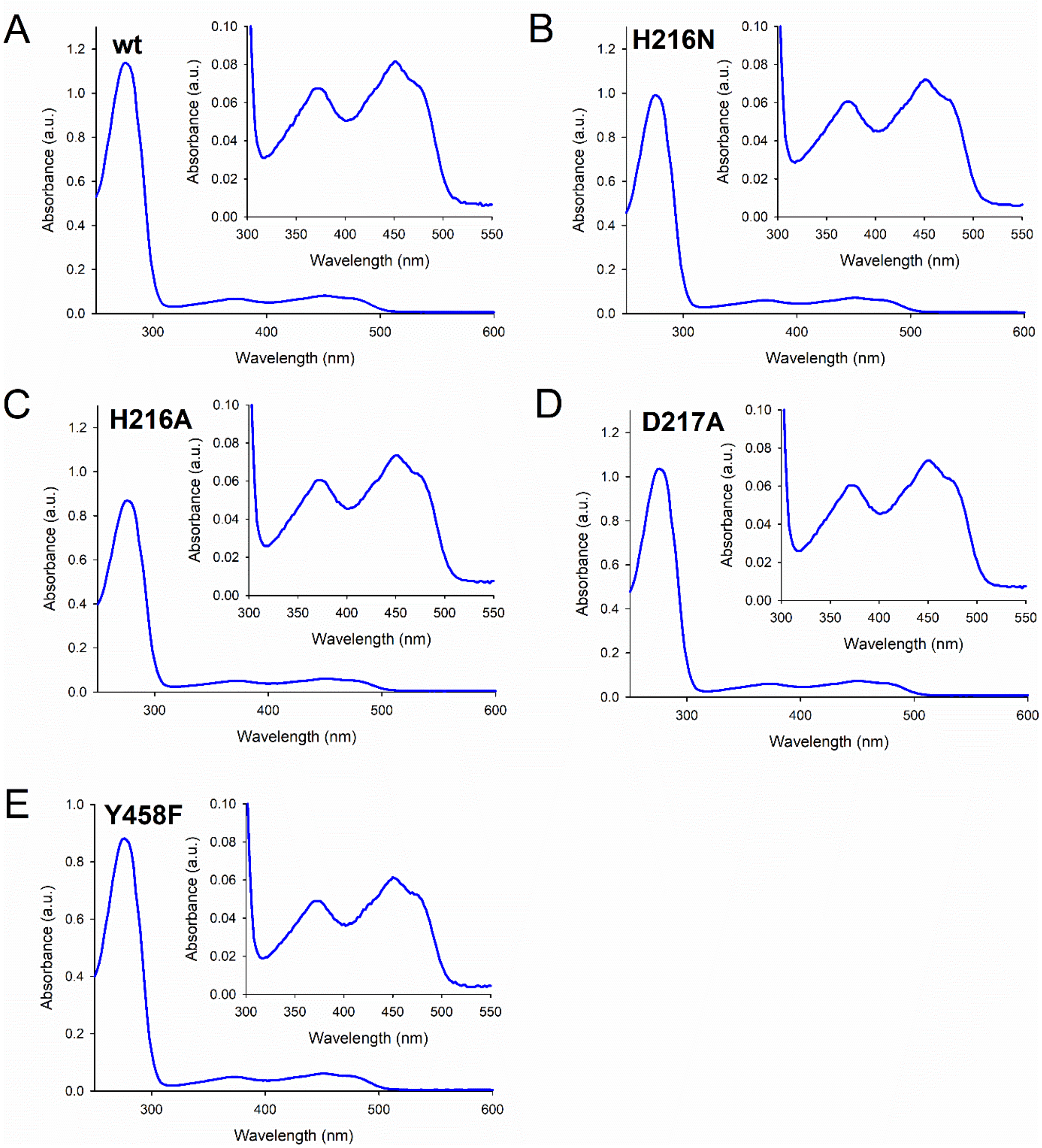

Figure S8. Absorption spectra of oxidized JsFMO (A) and variant enzymes H216N (B), H216A (C), D217A (D) and Y458F (E). The insets show an enhancement of the FAD absorbance. Spectra were recorded on a wellplate reader (XXX).

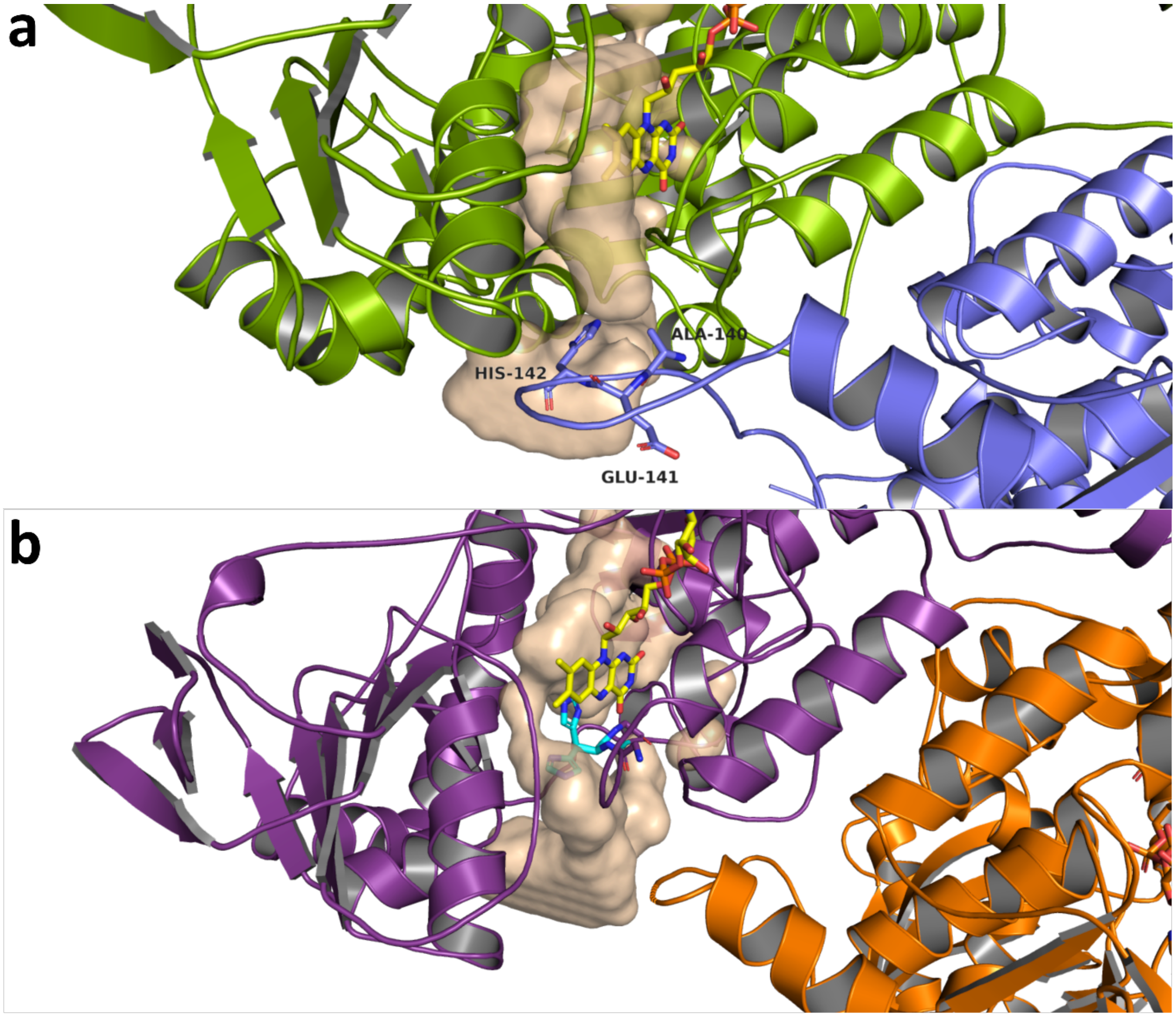

Figure S9. The final part of the tunnel in the dimer AC (a) and BD (b). a) Chain C (green) was used to calculate the tunnel since chain A (blue) shows the density for all the residues in the loop. The residues blocking the tunnel are represented as sticks. b) Chain D is represented in purple, chain B in orange. His-216 in chain D is highlighted to show how the alternative conformation is obstructing the tunnel.

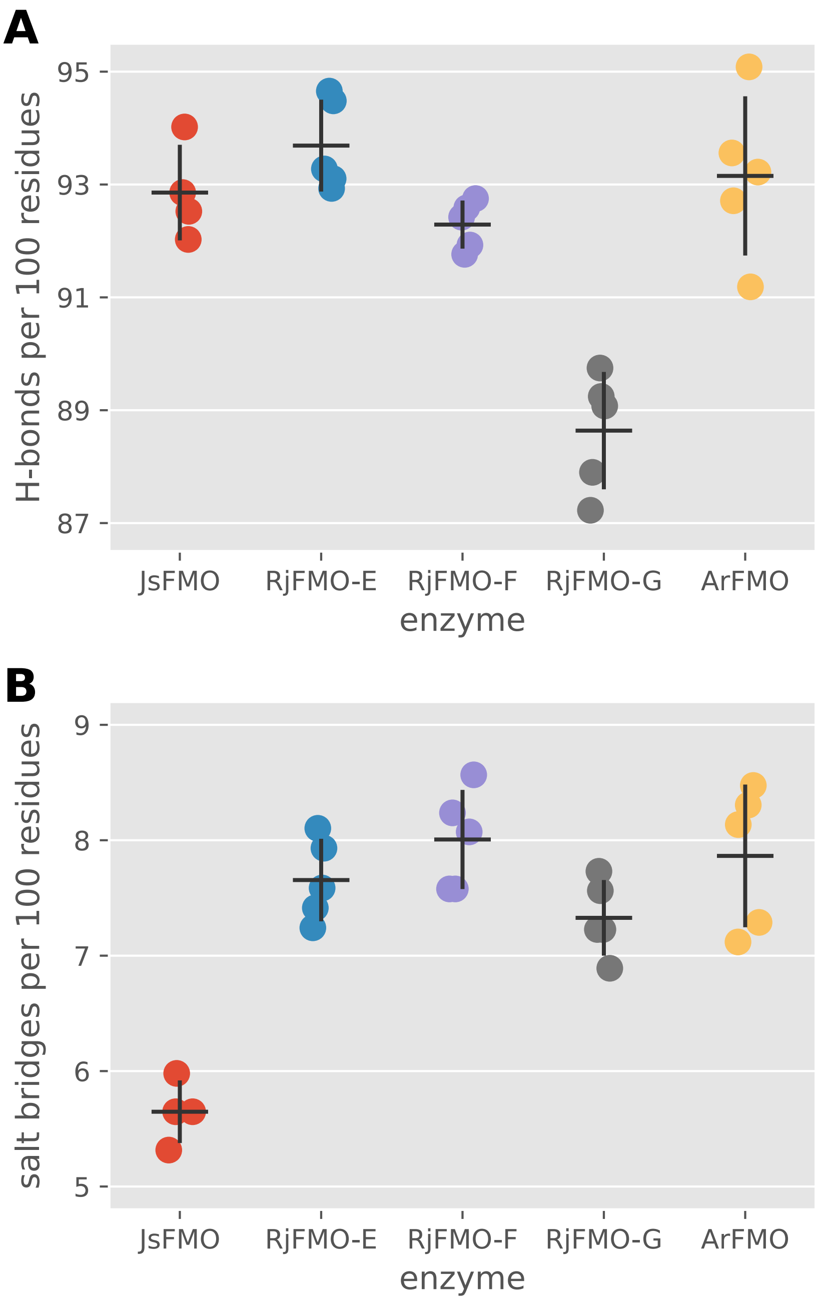

Figure S10. Comparison of polar interactions in different type II FMOs. Models of RjFMOs and ArFMO were generated using AlphaFold. Hydrogen bonds and salt bridges were analyzed using the program Hbplus v3.2.

JsFMO: FMO from *Janthinobacterium svalbardensis*, RjFMO…FMO from *Rhodococcus jostii*, ArFMO…FMO from *Actinomadura rubrobrunea*.

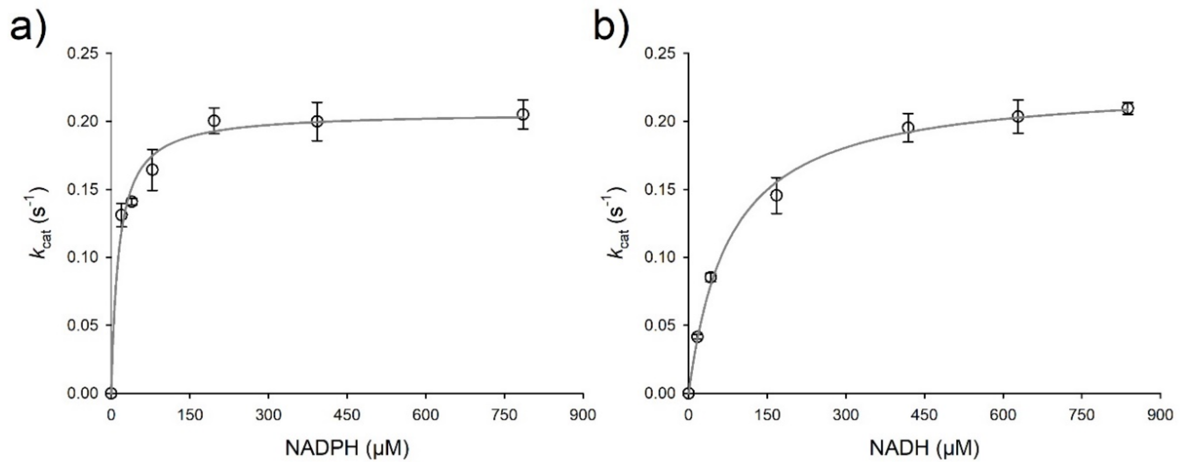

Figure S11. Kinetic constants for *Js*FMO with NADPH (a) and NADH (b). The parameters were determined measuring change in absorbance at 340 nm (ε_{340} = 6,220 M^{-1} cm^{-1}) using different concentrations of NADPH and NADH (20-900 μM) and 5 mM 1a. The K_M of *Js*FMO for NADPH was found at 14.4 ± 2.5 μM with a k_{cat} of 0.21 ± 0.1 s^{-1}, while the K_M for NADH was at 78.5 ± 7.5 μM with a k_{cat} of 0.23 ± 0.1 s^{-1}.

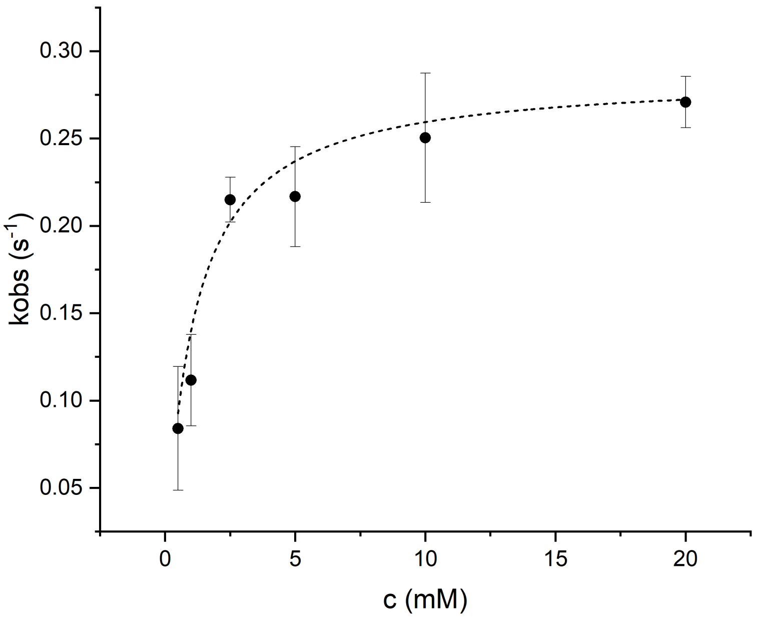

Figure S12. Kinetic parameters for JsFMO with **1a**. The parameters were determined measuring change in absorbance at 340 nm using different concentrations of **1a** (0.5-20 mM) and 0.25 mM of NADPH (ε_{340} = 6,220 M^{-1} cm^{-1}). Non-linear fit using Michaelis-Menten model was done using Origin 2019b software. k_{cat} = 0.29 ± 0.01 s^{-1}, K_M = 1.05 ± 0.23 mM.

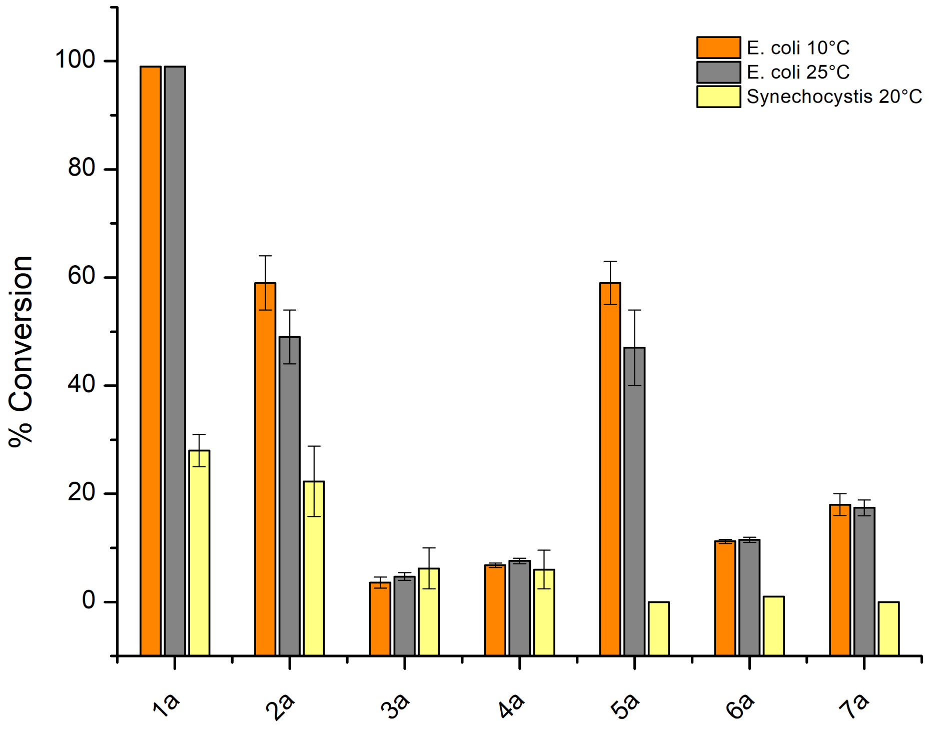

Figure S13. Conversions and specific activities obtained in whole cell biotransformations with *Synechocystis and E. coli*. Reactions were carried at 20°C with 5 mM of substrate.

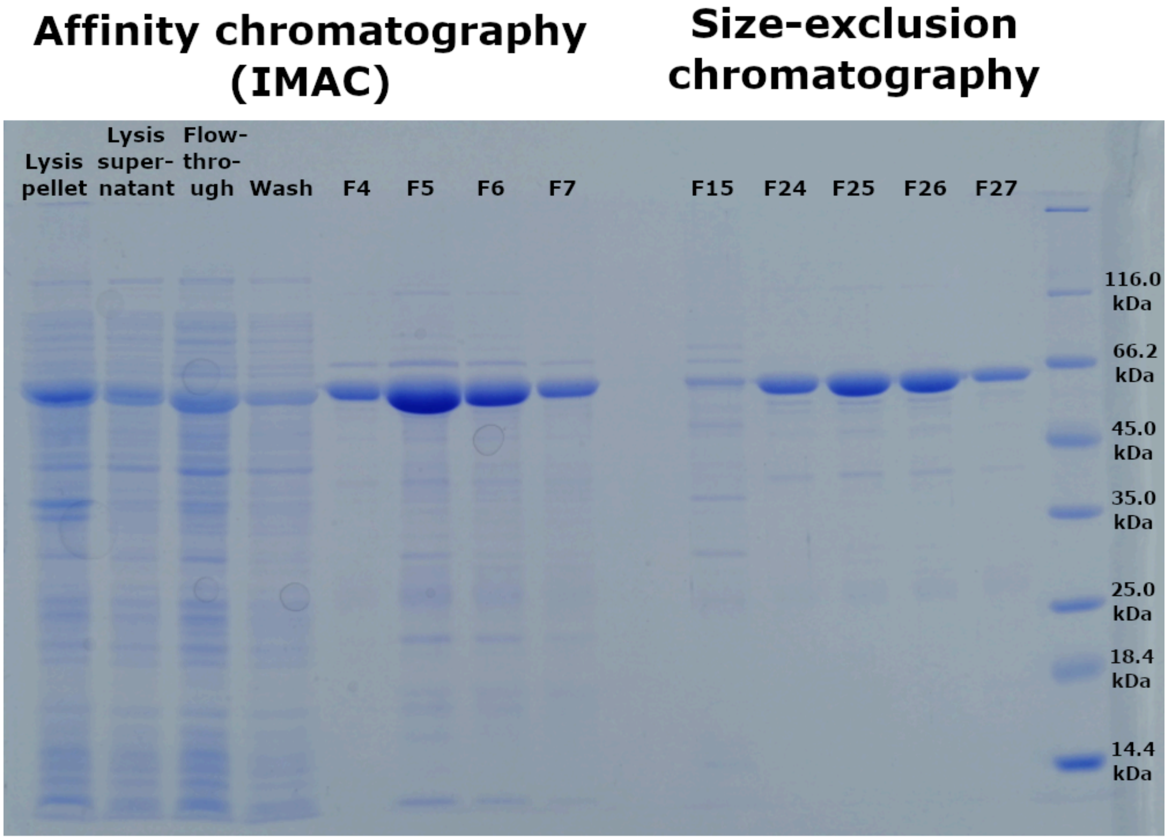

Figure S14. SDS gel of the purification of JsFMO. F4-F7 are the elution fractions used for the activity characterization and the kinetic analysis. F24-F27 are the elution fractions used for the protein crystallization

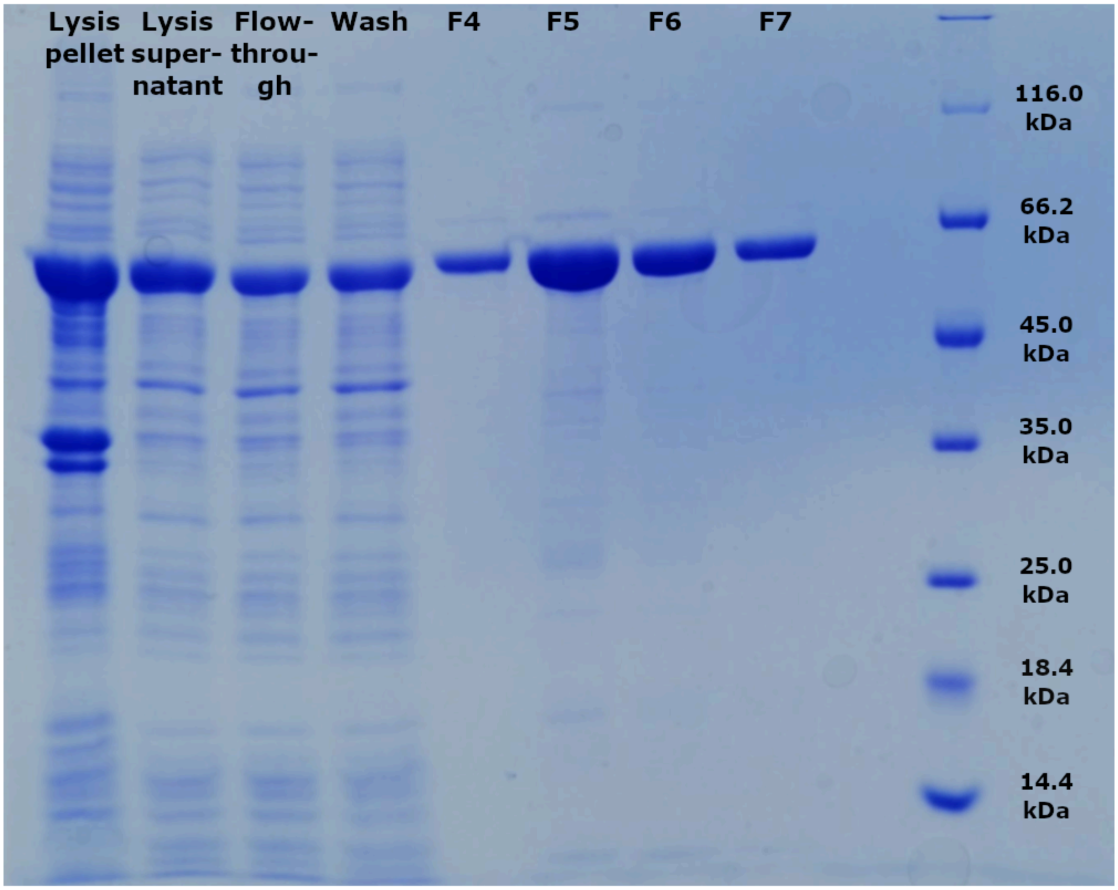

Figure S15. SDS gel of the Immobilized Metal affinity-chromatography purification of JsFMO H216A.

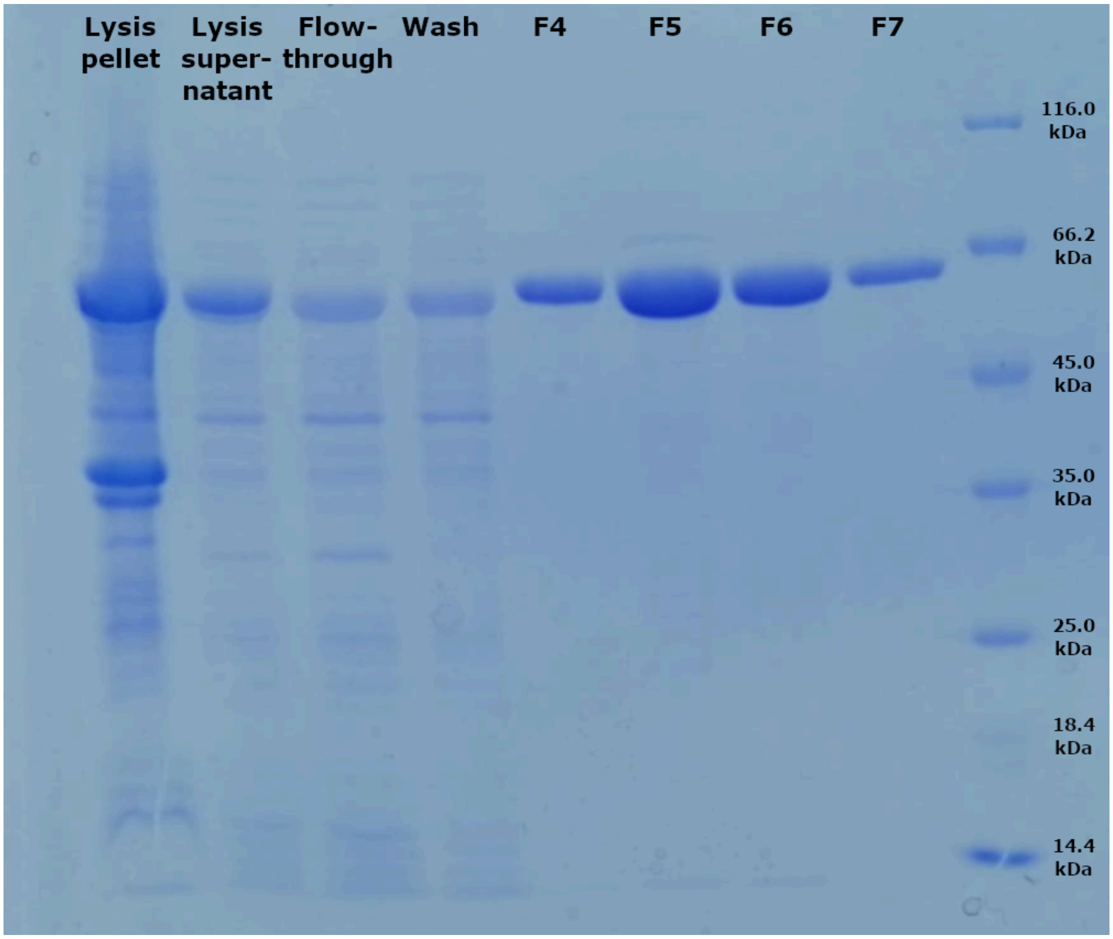

Figure S16. SDS gel of the Immobilized Metal affinity-chromatography purification of JsFMO H216N.

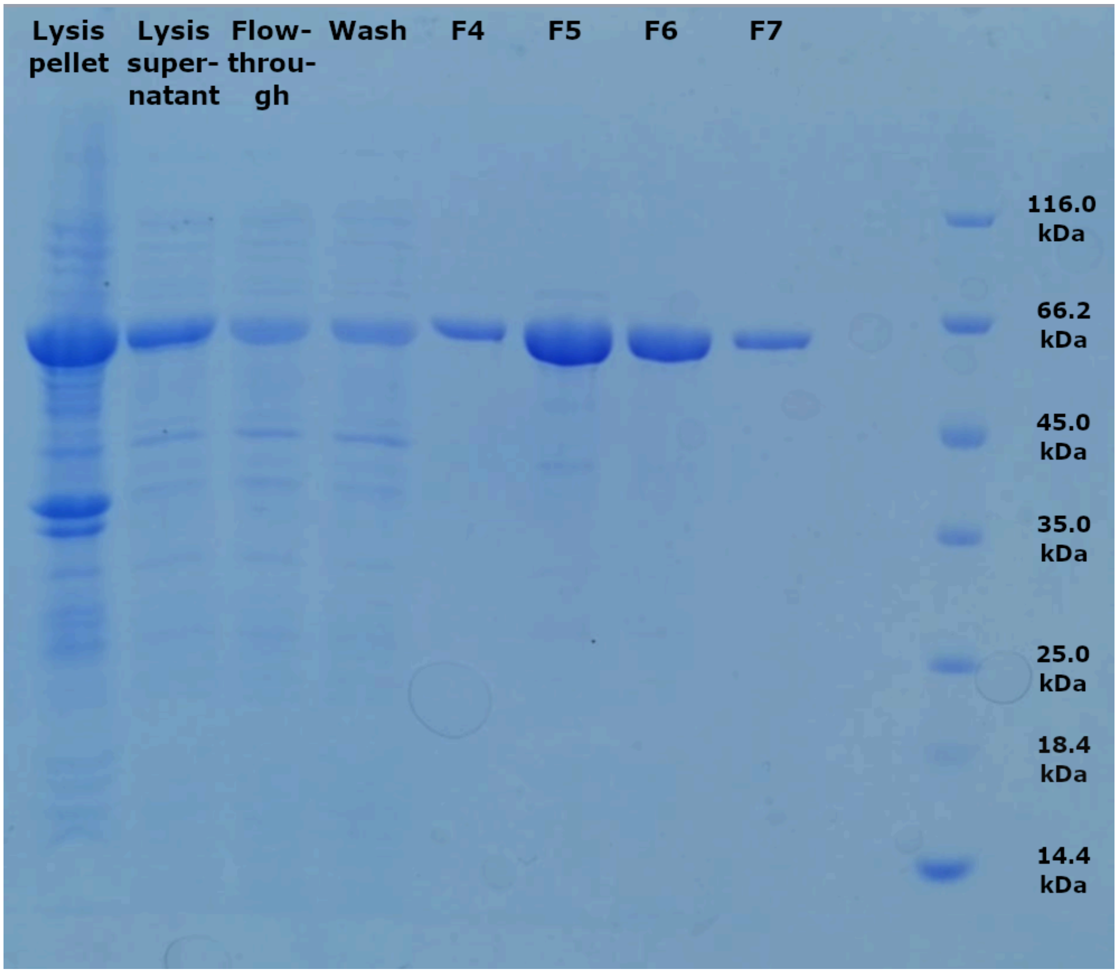

Figure S17. SDS gel of the Immobilized Metal affinity-chromatography purification of JsFMO Y458F.

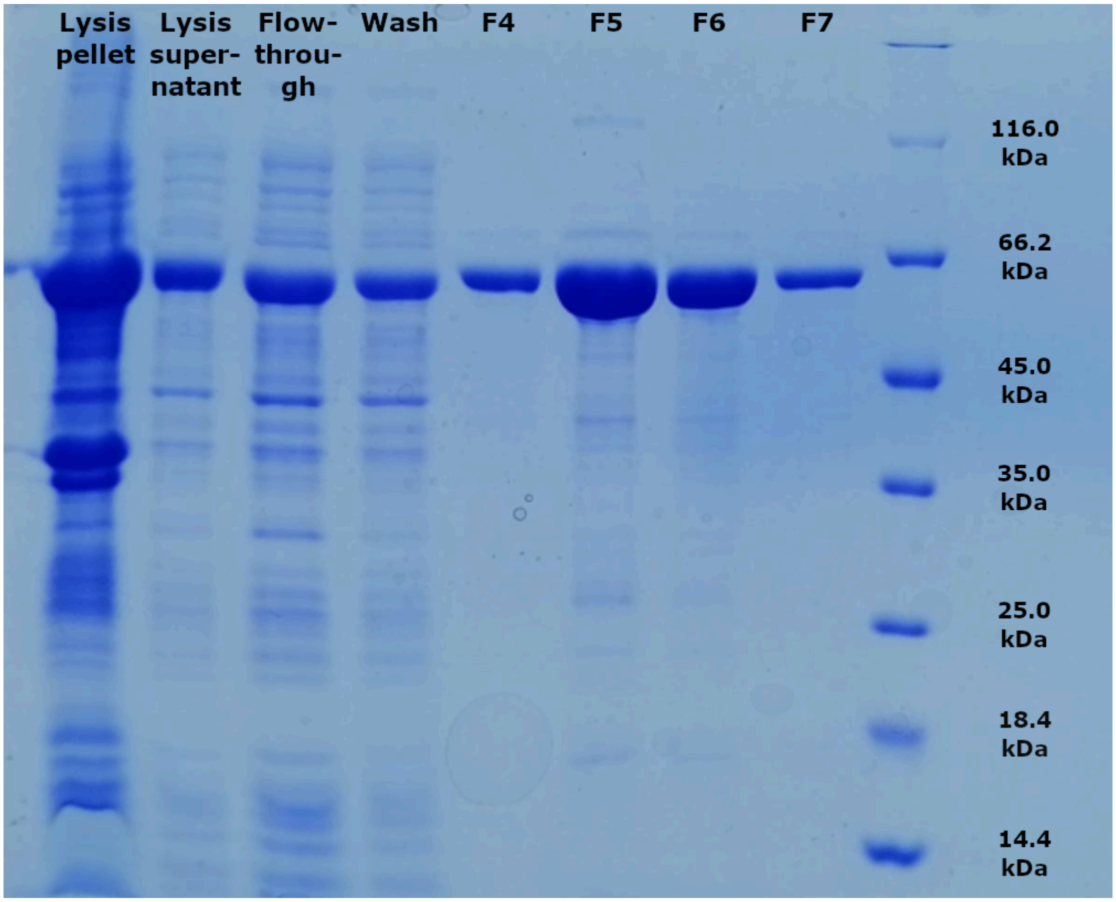

Figure S18. SDS gel of the Immobilized Metal affinity-chromatography purification of JsFMO D217A.

Table S1. Aminoacidic sequences of the enzymes used to construct the phylogenetic tree.

Name	Amino Acidic Sequence
JsFMO	MQTDSNPHDLAVAGILEQLEGCLRASDSTGAAQLFEPDGYWRDLVLFTWNLKTLEGREQIAAMLAAQLGAVQPVSIR<br>IADGEHAVEAGGVLQSWITVETNVARGVGFIRIRDGKIWTLLTTMSELKGFEEAKGGRRPMGAEHGARTDRSSWLEQ<br>REQEAKELGYARQPYCVIIGGGQGGIALGARLRQLNVPTIIIEKNARPGDSWRKRYKSLCLHDPVWYDHMPYIPFPDN<br>WPVFTPKDKVGDWLEMYTKVMELNYWGSTSCESASFDAASGEWTVQVLRDGQPVTLKPKQLVLATGMSGKANMP<br>KFKGMDVFQGEQQHSSQHPGPDAYAGKKVVVVGANNSAHDICAALWEAGVDVTMVQRSSTHIVKSDSLMDLALG<br>DLYSERALAAGMTTNKADLTFASIPYKILANFQKPVFKAIRERDADFYARLEERGFMLDFGDDDSGLFMKYLRRGSGYYI<br>DVGASELVAEGKIKLKSGVGVQELKSHSIVLSDGTELPADLVVYATGYGSMNGWAADLISPEVANKVGKVWGLGSATT<br>KDPGPWEGEQRNMWKPTQQQALWFHGGNLHQSRHYSQYLSLQLKARMEGLNTPVYGQQEVHHLS
PsFMO B	MTQLDDRPVTAADTTEALDPATAWFAAFEDALTARDVDRAAGLFAATSFWRDLIAFTWNLTTVENPDGVAGLLTSTL<br>DRVDPRGFRLTEPAATADGVTTAWFEFETSVGRGRGLVRIVDEDGPKAWTFLTTLYELTGHEEPRGTRRPMGAEHGA<br>TKERVTWLEKRQAEDAALGVDTQPYVLVVGGGQGGIALGARLRQLGVPALVIDKHPRPGDQWRNRYKSLCLHDPVW<br>YDHLPYLKFPDNWPVFAPKDKVGDWLEFYTRVMEVPYWSSTVATDASYDEETGEWTVHLEREGKPLVLKPTHLVMA<br>TGMSGKPNIPVVPGADVFQGEQHHSSQHPGPDAYAGKRVVVIGSNNSAFDICGALWETGADVTMVQRSSTHIVKSD<br>TLMDIGLGDLYSERAVEAGMTTEKADLVFASLPYRIMHEFQIPLYDQMRERDKDFYDRMEAAGFDLDWGDDGSGLF<br>MKYLRRGSGYYIDVGAAELVASGDVKLAHGQVDHLTETAVVLEDGTELPADLVVYATGYGSMNGWAADLISQEVAD<br>RVGKVWGLGSDTTKDPGPWEGEQRNMWKPTQQENLWFHGGNLHQSRHYSLYLALQLKARHAGIETPVHGLQEVH<br>HLG
PsFMO C	MGTQPQTFDEATLREAIESANLPTLLMVMFQLTGDRRWIAEPYRPKRAKGMSDNDTGGFAPEIQDEVRAAAYDILQE<br>WRGGRPVAVPLPDDEMLVELIATCVGEEVPSEFGPMFAEDMRASLEGDRPHPHVEDGAVLSVIIVGAGISGLAAAVEL<br>NAAGIRATIFEKNPDVGGTWWENRYPGCGVDTPSHVYSLSYQPRRWSTYYGKRDEVLDYVRDVARKTGVVDQVQFE<br>TAVESAVWDEGTQRWTVTTRTAEGVVSEHVANVVITAVGQLNRPAVPAIPGAETFTGRQFHSAEWPEGFDVAGLRV<br>GVVGSGASAMQIVPAVADRVSSLTVFQRSPQWIAPSFNYSSPVPEPVHWLMDNVPNYRLWYRLRLSWLINDRLYPSL<br>EIDPAWPHPERSINAHNDGHRRALTRYIQEELAGRDDLLEKSLPTYPPFGKRMLIDNGWYAALRKEGVELVTDGVASID<br>ETGLTTTAGQHVELDVIVYATGFEAKKMLYPMDIRGRDGVSIRDRWGDEDAKAYLGLTVPDFPNLMVMYGPNLNLG<br>HGGSYMFAGECQARYIAQMCALLTERHVASFEVRRDVHDDYNQRVDAQHARMIWSHRGMDTWYRNSAGRIVTNS<br>PWRVVDYWNMTRTVNPDDFVLAPAAEGELVEAGS
PsFMO A	MKVDTGSSPDEVKAAFEAWLADFADALATADTGATTDLLDAGCWWRDLLALSWDLGTYHGTDRIAGLLDEHLKPAQ<br>VSGVRVVTEFGPRFVAEEGGSGTIEGFFTFETAGAWCRGVARLRQGDDGWRAWTVMTGVEDLKGHERALGERRPT<br>GPRHEVGATTQRNWKDKREAAQAYDDREPEVVILGAGQGGLALAANLRLMGVDALILEKSARIGDGWRRRYHSLVL<br>HDPVWADHLPYLPFPQSWPIYSPKDKIADWFEFYAQAMELNVWCSAEMTDAAYDEQAGAWTLTVRTEQGERTLRP<br>RNVVLATGAAGEPNIPDFPGRDEFAGTVYHSSQHGAGGSWAGKKAIVVGACNSGHDIAQDLHEAGAEVTLVQRSST<br>HIISQQHGIPAIFGANFTESGPPTEYADLLASAFPWPLVLEAAKEGVKQTAEKDAELLASLEAVGFQLNDGPDGTGLMG<br>FALAKGGGYYIDVGASGLIASGRIALAQGSGLAEFTPSGIRLADGRELDADLVVLATGYSNMRETARRLFGDGVADRLP<br>SVLGIGEDGEIGGLYRRTGQPGFWFMGGPLAWVRVYSKHLALQITADLNGVRS
FMO E	MTTTFSDTDLRTDAQAWLDGFSRFLAAELAPTAVFAPQAYWRDVLAFTGDLRTFSDEIPAELLRRQELTKATNIRIAED<br>RTPPRLVERAGIPCLEVIFEFDTLAGSAVGVARLVDVPERGLLVRSLFTTLDQLADHPERTGEHRPVGQADSSKFGGPN<br>WLDRRIAAQAYENRDPDVLIVGGGQSGLTLAARLGQLDVDALVVDTHARPGDNWRTRYHALTLHNAVWLNDLPYM<br>PFPATWPQFVPKDKLAGWFEAYVEAMEINFWGTTAFIGGDYDEQSQSWVARVRRGDGTVRTLRPKHVVIATGVSGI<br>PYVPELPGLSQFAGRTLHSSEYDDANDFAGQRVVIIGTGNSAHDVAQDLHAHGIDVTMVQRSSTTIVSVDPSAAAADA<br>SYLTAPTLEDCDLLSMATVYPDLYTGSQMITATMKELDKDLVAALNRIGFRTDYGEEDTGQQMKFMRRGGGYYLNVG<br>CSDLLISGQVGLVQYADTAGFVAEGLSLTNGDVVEADAVILATGYQTQQEGVRALLGDEIADAVGPIWGYDDEGEVR<br>NTWRRTAQPGLWFSSGNFQLCRIYSKVLAMQIRTELDNG
FMO F	MTQTVQPAAVQTSLTPQERVDLWLASFESALAARDVDRAAGMFAVDSFWRDLVAFTWNLKTVEGRDAVAAMLHA<br>RLDDTDPVNFRTTETPDEADGVTSAWIEFETATGRGKGHLRLKGDEAWTFLTTMQELKGHEERRGRNRVKGAVHGS<br>GGDTLSWAEKREIEERELGYTRQPYVLVIGGGQGGIALGARLRQLGVPAIVVDKNERPGDQWRNRYKSLCLHDPVWY<br>DHLPYMPFPDNWPVFAPKDKIGDWLEMYTKVMEIPYWSSTTCTSATFDDETKEWTVVLDRDGEDVVLHPKQLVLAT<br>GMSGKPNVPSFPGQDVFRGEQHHSSRHPGPDAYVGKRVVVVGANNSAHDICKALFENGADVTMLQRSSTHIVKSDS<br>LMDLGLGDLYSERAVAAGMTTEKADLTFASLPYKIMHEFQIPIYQKIAERDRDFYDRLEKAGFKLDFGDDGSGLFMKYL<br>RRGSGYYIDVGASELVADGSIHLVSGQVDHLTEDAVVLTDGTELPADLVVYATGYGSMNGWAADLMGQEVADKVGK<br>CWGLGSDTTKDPGPWEGEQRNMWKPTQQEALWFHGGNLHQSRHYSLYLALQLKARHEEIPTPVYGLQEVHHLS

FMO G	MTATLDAPVDTNIPQPGDIARRWLAGFGATLERGDARGAAQHFLVDGWWRDLLSFTWDLHTTHGRADIESRLADSV<br>PVHEPRHLVLSPAHPAEAVADPEGDWIQAFFTFETTLARSRGFVRLRRDDDGEWRAWTLISAMEEIKGHEEKKGHRR<br>VQGTNHGAHRGKINWLDRRTAKGEFETEQPAVVIVGAGQGGLALAARLGQLGVDTLLVERNDRIGDSWRKRYHSLV<br>LHDPVWYDHLPYLNFPDHWPVFTPKDKLANWFEFYADAMELNVWTGTEFTGGSYDDATGEWTVTVARDDGSTRTL<br>HPRHVVLATGMSGVPNIPRIAGADTFEGTIEHSSWFVGGREMQGKKALVVGCCNSGHDIAQELNEQGADVTILQRSS<br>TYVMSSKHGIPGLFGGVYEEGGPAVQDADLIFASLPYPLLAGIHAGATEAIAEKDAEMLDGLRKAGFKVDFGEDGSGLF<br>MKYLRRGGGYYIDVGASELIASGEVSVKQGTEIDHFTPDGVVFADGTEMPVDVVVLATGYKNMRESARKFLGDAVAD<br>RCQDVWGLDAEGELRTVWRRSGHPGFWFMAGNLHQSRHYSKYLAFQIKAQEEGLQPIR
PAMO	MAGQTTVDSRRQPPEEVDVLVVGAGFSGLYALYRLRELGRSVHVIETAGDVGGVWYWNRYPGARCDIESIEYCYSFSE<br>EVLQEWNWTERYASQPEILRYINFVADKFDLRSGITFHTTVTAAAFDEATNTWTVDTNHGDRIRARYLIMASGQLSVP<br>QLPNFPGLKDFAGNLYHTGNWPHEPVDFSGQRVGVIGTGSSGIQVSPQIAKQAAELFVFQRTPHFAVPARNAPLDPE<br>FLADLKKRYAEFREESRNTPGGTHRYQGPKSALEVSDEELVETLERYWQEGGPDILAAYRDILRDRDANERVAEFIRNKI<br>RNTVRDPEVAERLVPKGYPFGTKRLILEIDYYEMFNRDNVHLVDTLSAPIETITPRGVRTSEREYELDSLVLATGFDALTG<br>ALFKIDIRGVGNVALKEKWAAGPRTYLGLSTAGFPNLFFIAGPGSPSALSNMLVSIEQHVEWVTDHIAYMFKNGLTRSE<br>AVLEKEDEWVEHVNEIADETLYPMTASWYTGANVPGKPRVFMLYVGGFHRYRQICDEVAAKGYEGFVLT
CPMO	MTTMTTMTTEQLGMNNSVNDKLDVLLIGAGFTGLYQLYHLRKLGYKVHLVDAGADIGGIWHWNCYPGARVDTHCQ<br>IYQYSIPELWQEFNWKELFPNWAQMREYFHFADKKLDLSKDISFNTRVQSAVFDEGTREWTVRSIGHQPIQARFVIAN<br>LGFGASPSTPNVDGIETFKGQWYHTALWPQEGVNMAGKRVAIIGTGSSGVQVAQEAALDAKQVTVYQRTPNLALP<br>MHQKQLSAEDNLRMKPELPAAFERRGKCFAGFDFDFIAKNATELSAAERTEILEELWNAGGFRYWLANFQDYLFDDK<br>ANDYVYEFWRDKVRARIKDPKVAEKLAPMKKPHPYGAKRPSLEQWYYEIFNQNNVTLVDVNETPVLRITEKGIVTAEG<br>EAEFDLIVFATGFDAVTGGLTSIDFRNNQGQSFKDVWSDGIRTQLGVATAGFPNLLFGYGPQSPAGFCNGPSSAEYQG<br>DLLIQLMNYLRDNNISRIEAQSEAQEEWSKLIADFWDSSLFPRAKSWYQGSNIPGKKVESLNFPLGLPTYISKFNESAEK<br>GYAGFSLAS
HAPMO	MSAFNTTLPSLDYDDDTLREHLQGADIPTLLLTVAHLTGDLQILKPNWKPSIAMGVARSGMDLETEAQVREFCLQRLID<br>FRDSGQPAPGRPTSDQLHILGTWLMGPVIEPYLPLIAEEAVTAEEDLRAPRWHKDHVASGRDFKVVIIGAGESGMIAAL<br>RFKQAGVPFVIYEKGNDVGGTWRENTYPGCRVDINSFWYSFSFARGIWDDCFAPAPQVFAYMQAVAREHGLYEHIRF<br>NTEVSDAHWDESTQRWQLLYRDSEGQTQVDSNVVVFAVGQLNRPMIPAIPGIETFKGPMFHSAQWDHDVDWSGK<br>RVGVIGTGASATQFIPQLAQTAAELKVFARTTNWLLPTPDLHEKISDSCKWLLAHVPHYSLWYRVAMAMPQSVGFLE<br>DVMVDVGYPPTELAVSARNDRLRQDISAWMEPQFADRPDLREVLIPDSPVGGKRIVRDNGTWISTLKRDNVSMIRQP<br>IEVITPKGICCVDGTEHEFDLIVYGTGFHASKFLMPINVTGRDGVALHDVWKGDDARAYLGMTVPQFPNMFCMYGPN<br>TGLVVYSTVIQFSEMTASYIVDAVRLLLEGGHQSMEVKTPVFESYNQRVDEGNALRAWGFSKVNSWYKNSKGRVTQN<br>FPFTAVEFWQRTHSVEPTDYQLG
CHMO	MSQKMDFDAIVIGGGFGGLYAVKKLRDELELKVQAFDKATDVAGTWYWNRYPGALTDTETHLYCYSWDKELLQSLEI<br>KKKYVQGPDVRKYLQQVAEKHDLKKSYQFNTAVQSAHYNEADALWEVTTEYGDKYTARFLITALGLLSAPNLPNIKGIN<br>QFKGELHHTSRWPDDVSFEGKRVGVIGTGSTGVQVITAVAPLAKHLTVFQRSAQYSVPIGNDPLSEEDVKKIKDNYDKI<br>WDGVWNSALAFGLNESTVPAMSVSAEERKAVFEKAWQTGGGFRFMFETFGDIATNMEANIEAQNFIKGKIAEIVKD<br>PAIAQKLMPQDLYAKRPLCDSGYYNTFNRDNVRLEDVKANPIVEITENGVKLENGDFVELDMLICATGFDAVDGNYVR<br>MDIQGKNGLAMKDYWKEGPSSYMGVTVNNYPNMFMVLGPNGPFTNLPPSIESQVEWISDTIQYTVENNVESIEATK<br>EAEEQWTQTCANIAEMTLFPKAQSWIFGANIPGKKNTVYFYLGGLKEYRSALANCKNHAYEGFDIQLQRSDIKQPANA
OTEMO	MSNRAKSPALDAVVIGAGVTGIYQAFLINQAGMKVLGIEAGEDVGGTWYWNRYPGCRLDTESYAYGYFALKGIIPEW<br>EWSENFASQPEMLRYVNRAADAMDVRKHYRFNTRVTAARYVENDRLWEVTLDNEEVVTCRFLISATGPLSASRMPDI<br>KGIDSFKGESFHSSRWPTDAEGAPKGVDFTGKRVGVIGTGATGVQIIPIAAETAKELYVFQRTPNWCTPLGNSPMSKEK<br>MDSLRNRYPTILEYVKSTDTAFPYHRDPRKGTDVSESERDAFFEELYRQPGYGIWLSGFRDLLLNKESNKFLADFVAKKIR<br>QRVKDPVVAEKLIPKDHPFGAKRVPMETNYYETYNRDNVHLVDIREAPIQEVTPEGIKTADAAYDLDVIIYATGFDAVT<br>GSLDRIDIRGKDNVRLIDAWAEGPSTYLGLQARGFPNFFTLVGPHNGSTFCNVGVCGGLQAEWVLRMISYMKDNGFT<br>YSEPTQAAENRWTEEVYADFSRTLLAEANAWWVKTTTKPDGSVVRRTLVHVSGGPEYRKRCEQVAYNNYNGFELA
CPDMO	MSQLIQEPAEAGVTSQKVSFDHVALREKYRQERDKRLRQDGQEQYLEVAVTCDEYLKDPYADPIVRDPVVRETDVFIIG<br>GGFGGLLAAVRLQQAGVSDYVMVERAGDYGGTWYWNRYPGAQCDIESYVYMPLLEEMGYIPTEKYAFGTEILEYSRS<br>IGRKFGLYERTYFQTEVKDLSWDDEAARWRITTDRGDKFSARFVCMSTGPLQRPKLPGIPGITSFKGHSFHTSRWDYSY<br>TGGDQTGNLEGLKDKRVAIIGTGATSIQAVPHLAAYAQELYVIQRTPISVGFRGNKPTDPEWAKSLQPGWQQARMDN<br>FNAITHGMPVDVDLVQDSWTKIFGEIGVFLGSDGSRAQMVDFQLMEQIRARVDQEVKDPATAESLKPYYNIMCKRP<br>GFHDSYLPSFNKPNVTLVDTQGAGVERITEKGLVVNGREYEVDCLIYATGFEYQTKLSRRNGYEIHGRNGQPLSDKWK<br>DGLSTLWGYHIRDFPNCFILGNGQSAVTPNFTHMLNEAGKHVAYVVKHCLDERVDVFEPTAEAEQAWVDHVMSFA<br>GIKQQYDRECTPSYYNNEGQVNDVALTRNNFYPGGAVAFINILREWREKGDFAQFQQRKR

STMO	MNGQHPRSVVTAPDATTGTTSYDVVVVGAGIAGLYAIHRFRSQGLTVRAFEAASGVGGVWYWNRYPGARCDVESID<br>YSYSFSPELEQEWNWSEKYATQPEILAYLEHVADRFDLRRDIRFDTRVTSAVLDEEGLRWTVRTDRGDEVSARFLVVAA<br>GPLSNANTPAFDGLDRFTGDIVHTARWPHDGVDFTGKRVGVIGTGSSGIQSIPIIAEQAEQLFVFQRSANYSIPAGNVP<br>LDDATRAEQKANYAERRRLSRESGGGSPHRPHPKSALEVSEEERRAVYEERWKLGGVLFSKAFPDQLTDPAANDTARA<br>FWEEKIRAVVDDPAVAELLTPKDHAIGAKRIVTDSGYYETYNRDNVELVDLRSTPIVGMDETGIVTTGAHYDLDMIVLA<br>TGFDAMTGSLDKLEIVGRGGRTLKETWAAGPRTYLGLGIDGFPNFFNLTGPGSPSVLANMVLHSELHVDWVADAIAYL<br>DARGAAGIEGTPEAVADWVEECRNRAEASLLNSANSWYLGANIPGRPRVFMPFLGGFGVYREIITEVAESGYKGFAILE<br>G
ACMO	MSTTTLDAAVIGTGVAGLYELHMLREQGLEVRAYDKASGVGGTWYWNRYPGARFDSEAYIYQYLFDEDLYKGWSWS<br>QRFPGQEEIERWLNYVADSLDLRRDISLETEITSAVFDEDRNRWTLTTADGDTIDAQFLITCCGMLSAPMKDLFPGQSD<br>FGGQLVHTARWPKEGIDFAGKRVGVIGNGATGIQVIQSIAADVDELKVFIRTPQYALPMKNPSYGPDEVAWYKSRFGE<br>LKDTLPHTFTGFEYDFTDAWEDLTPEQRRARLEDDYENGSLKLWLASFAEIFSDEQVSEEVSEFVREKMRARLVDPELC<br>DLLIPSDYGFGTHRVPLETNYLEVYHRDNVTAVLVRDNPITRIRENGIELADGTVHELDVIIMATGFDAGTGALTRIDIRG<br>RDGRTLADDWSRDIRTTMGLMVHGYPNMLTTAVPLAPSAALCNMTTCLQQQTEWISEAIRHLRATGKTVIEPTAEGE<br>EAWVAHHDELADANLISKTNSWYVGSNVPGKPRRVLSYVGGVGAYRDATLEAAAAGYKGFALS
CDMO	MTTSIDREALRRKYAEERDKRIRPDGNDQYIRLDHVDGWSHDPYMPITPREPKLDHVTFAFIGGGFSGLVTAARLRESG<br>VESVRIIDKAGDFGGVWYWNRYPGAMCDTAAMVYMPLLEETGYMPTEKYAHGPEILEHCQRIGKHYDLYDDALFHT<br>EVTDLVWQEHDQRWRISTNRGDHFTAQFVGMGTGPLHVAQLPGIPGIESFRGKSFHTSRWDYDYTGGDALGAPMD<br>KLADKRVAVIGTGATAVQCVPELAKYCRELYVVQRTPSAVDERGNHPIDEKWFAQIATPGWQKRWLDSFTAIWDGVL<br>TDPSELAIEHEDLVQDGWTALGQRMRAAVGSVPIEQYSPENVQRALEEADDEQMERIRARVDEIVTDPATAAQLKA<br>WFRQMCKRPCFHDDYLPAFNRPNTHLVDTGGKGVERITENGVVVAGVEYEVDCIVYASGFEFLGTGYTDRAGFDPTG<br>RDGVKLSEHWAQGTRTLHGMHTYGFPNLFVLQLMQGAALGSNIPHNFVEAARVVAAIVDHVLSTGTSSVETTKEAEQ<br>AWVQLLLDHGRPLGNPECTPGYYNNEGKPAELKDRLNVGYPAGSAAFFRMMDHWLAAGSFDGLTFR
MEKMO	MSAQSKLAAGSCAYGNVTSLDAMVIGAGVAGLYQLYRLREMGLTVRAYDTASGVGGTWYWNRYPGARFDSQAEIY<br>QYWFSEELYKSWQPTERFPAQPETEEWLNFVANRLNLKKDIQFNTRIASAHFCEDSGRWVVTTAAGETINTQYLISCC<br>GMLSAPLSDRFPGQADFQGQIYHTGLWPKDPVDFNGKRVAVVGTGATGIQVIQTIAPTVGSMTVFVRTPQYVIPMR<br>NPKYSKADWEKWGTQFHQLKKRVRETFAGFDYDFDAGPWAEKTPDERQAVLEQLWKDGSLAMWLASFPEMFFDE<br>QVNEVVSQFVRIKMRERLRSRPDLCDLLIPTDYGFGTHRVPLENNYLEVYLQSNVKAVDCKQSPIERIVPQGIQTADGKI<br>HEVDIIVLAVGFDAGSGALSRIDIRGRDSRSLKEQWQQEIRTAMGLQIHGYPNLFTTGAPLAPSAALCNMTTCLQQQV<br>DWITGCIEFAAEHGKHVVEASKALEDNWVQHHDETAAKTLVVKTDSWYMGSNVDGKPRRLLSYIGGAGDYHRRCAE<br>IAAQGYPGFEMA
SMFMO	MDSVDVVVIGGGQSGLSAGYFLRRSGLSYVILDAEASPGGAWQHAWHSLHLFSPAGWSSIPGWPMPASQGPYPAR<br>AEVLAYLAQYEQKYALPVLRPIRVQRVSHFGERLRVVARDGRQWLARAVISATGTWGEAYTPEYQGLESFAGIQLHSA<br>HYSTPAPFAGMRVAIIGGGNSGAQILAEVSTVAETTWITQHEPAFLADDVDGRVLFERATERWKAQQEGREPDLPPG<br>GFGDIVMVPPVLDARARGVLAAVPPPARFSPTGMQWADGTERAFDAVIWCTGFRPALSHLKGLDLVTPQGQVEVD<br>GSGLRALAVPSVWLLGYGDWNGMASATLIGVTRYAREAVRQVTAYCADHQDR
CFMO	MDTPVMDSTDTDSTDIVIIGGGQAALSVAYYLRRSKYSFVMLDAEQTPGGAWLHGWDSLRLFSPSTWSSLSGWQMP<br>PTGETYPSRDQVVDYLRHYESRYEFPVQRPVWVSAVNNLGDRLEVVSERQQWRARVVISATGTWRNPFIPAYPGADL<br>FQGAQLHSAHYQSPAPFAGQKVLVVGGGNSGAQILAEVSRVADCTWVTTSEPIFLPDDVDGRVLFQRATDRWKAAQ<br>EGREIEQPVGGLGDVVMVPPVKEARERGALHAVRPFTRFTANGVVWADGTGSAVDAVIWCTGFRPALAHLQSLGVI<br>NPDGKVDLAGTRSLQEPRLWLLGYGEWTGLASATLIGVGRSARATAEEIIQYLDSA
PSFMO	MPPILDVIVIGGGQAALTTAYFLRRTSLSYLLLDEQPGPGGAWLHAWDSLRLFSPAAWSSIAGWPMPSPTEPGNPTRN<br>DVIDYLRRYEDRYQFPIQRPVRVDTVTRLDDLWRVQAGDQQWLARAVISATGTWSKPFIPPYEGRELFQGAQIHSAHY<br>RTPAPFAGKRVMVVGGGNSGAQVLAELSSVSETLWITQEPPAFLPDEVDGRVLFERATARWKAQQEGRSIDEPAGGF<br>GDIVMVPPVREARERGVLVAERPFARFTETGVEWADGRRENLDAVIWCSGFRPALDHLRELGVVEADGKVQVEDTR<br>VVKQPNLWLVGYGDWTGMASATLIGVTRTARSTADQVVQALTATPSRRP

**Table S2.** FAD loading and actvity of wild-type and variant FMOs. The FAD loading	**FAD loading**	**Spec. Act. (U mg^{-1})**	**Ratio**

was estimated from the absorbance spectra shown in Figure S9.	(%)	with 1a*	without 1a	(+1a/-1a)
**wild-type**	80.7	0.799 ± 0.016	0.021 ± 0.003	38
**H216N**	81.8	0.705 ± 0.012	0.018 ± 0.003	39
**H216A**	79.5	0.457 ± 0.015	0.028 ± 0.002	16
**D217A**	79.8	0.444 ± 0.043	0.018 ± 0.005	25
**Y458F**	78.5	0.418 ± 0.042	0.024 ± 0.015	17

* *bicyclo[3.2.0]hept-2-en-6-one, 5 mM; all reactions were carried out in triplicates*

Table S3. Set of sulphides, linear and cyclic ketones assayed for activity with JsFMO.

Name	CAS number
Methyl phenyl sulfide	100-68-5
Ethyl phenyl sulfide	622-38-8
Methyl p-tolyl sulfide	623-13-2
2-Chlorothioanisole	17733-22-1
4-Nitrothioanisole	701-57-5
Camphor	76-22-2
Norcamphor	497-38-1
(-)-Fenchone	7787-20-4
Bicyclo[3.2.0]hept-2-en-6-one	13173-09-6
Cyclobutanone	1191-95-3
Cyclopentanone	120-92-3
Cyclohexanone	108-94-1
Cyclooctanone	502-49-8
(+)-Carvone	2244-16-8
(-)-Carvone	6485-40-1
2-cyclopenten-1-one	930-30-3
2-methyl cyclohexanone	583-60-8
3-methyl cyclohexanone	591-24-2
4-methyl cyclohexanone	589-92-4
Propiophenone	93-55-0
α- tetralone	529-34-0
Valerophenone	1009-14-9
4-methyl propiophenone	5337-93-9
1-indanone	83-33-0
1-phenyl-2-butanone	1007-32-5
2-butanone	78-93-3
2-pentanone	107-87-9
2-octanone	111-13-7
2-heptanone	110-43-0
(-)-Verbenone	1196-01-6
Tropinone	532-24-1

Table S4. Parameters and retention times for gas chromatography analysis

	Program 1 (chiral)	Program 2 (chiral)	Program 3 (chiral)	Program 4 (chiral)	Program 5 (achiral)
**Column parameters**					
**Column**	ß-6TBDAc	Hydrodex-ß-6TBDM	Hydrodex-ß-6TBDM	Hydrodex-ß-6TBDM	ZB-5
**ID**	23254-3	23433-41	23433-41	23433-41	1003602
**Film Thickness**	0.25 µm	0.25 µm	0.25 µm	0.25 µm	0.25 µm
**Column Length**	50 m	25 m	25 m	25 m	30 m
**Inner Diameter**	0.25 mm ID	0.25 mm ID	0.25 mm ID	0.25 mm ID	0.32 mm ID
**Autosampler and Injection Port**					
**Injection volume**	1 uL	1 uL	1 uL	1 uL	1 uL
**Injection temperature**	230°C	230°C	230°C	230°C	230°C
**Carrier gas**	N_2	N_2	N_2	N_2	N_2
**Pressure**	118.4 kPa	79.9 kPa	79.9 kPa	79.9 kPa	35.8 kPa
**Total Flow**	103.5 mL min^{-1}	110.7 mL min^{-1}	110.7 mL min^{-1}	110.7 mL min^{-1}	24 mL min^{-1}
**Linear Velocity**	22.9 cm s^{-1}	30.1 cm s^{-1}	30.1 cm s^{-1}	30.1 cm s^{-1}	19.6 cm s^{-1}
**Purge Flow**	3 mL min^{-1}	3 mL min^{-1}	3 mL min^{-1}	3 mL min^{-1}	3 mL min^{-1}
**Split Ratio**	100	100	100	100	20
**FID detector**					
**Temperature**	250°C	250°C	250°C	250°C	320°C
**Sampling rate**	80 msec	40 msec	40 msec	40 msec	40 msec
**H2 flow**	32 mL min^{-1}	32 mL min^{-1}	32 mL min^{-1}	32 mL min^{-1}	40 mL min^{-1}
**Air flow**	200 mL min^{-1}	200 mL min^{-1}	200 mL min^{-1}	200 mL min^{-1}	400 mL min^{-1}
**Temperature program**					
Step 1					
**Rate (°C min^{-1})**	-	-	-	-	-
**Temperature (°C)**	60	80	80	80	60
**Hold Time (min)**	10	8	8	5	5
Step 2					
**Rate (°C min^{-1})**	3.5	3	3	3	10
**Temperature (°C)**	220	170	180	220	200
**Hold Time (min)**	2	0	0	2	-
Step 3					
**Rate (°C min^{-1})**	-	50	50		30
**Temperature (°C)**	-	220	220		300
**Hold Time (min)**	-	2	2	-	2
**Retention times (min)**					
**Substrates**	(*1R,5S*)-**1a**: 33.964	**2a**: 18.599	**3a**: 23.066	**(1R,5S)-5a**: 17.528	**1a**: 9.042
	(*1S,5R*)-**1a**: 34.780	-	-	**(1S,5R)-5a**: 17.899	**2a**: 12.071
	-	-	-	-	**3a**: 13.932

	-	-	-	-	**4a**: 3.624
	-	-	-	-	**5a**: 9.918
	-	-	-	-	**6a**: 7.719
	-	-	-	-	**7a**: 10.049
**Products**	(3*aR*,6*aS*)-**1b**: 49.853	(*S*)-**2b**: 35.384	**3b**(i): 39.703	**(1R,5S)-5b**: 31.917	**1b**: 13.991
	(3*aS*,6*aR*)-**1b**: 50.860	(*R*)-**2b**: 35.750	**3b**(ii): 40.094	**(1S,5R)- 5b**: 32.389	**2b**: 16.193
	-	-	-	-	**3b**: 17.878
	-	-	-	-	**4b**: 8.277
	-	-	-	-	**5b**: 14.918
	-	-	-	-	**6b**: 8.262
	-	-	-	-	**7b**: 10.53

Table S5. Statistics of X-ray data collection and refinement. Statistics for the highest-resolution shell are shown in parentheses.

Crystal structure of FMO from *Janthinobacterium svalbardensis*	
Wavelength	1.34
Resolution range	48.25 - 2.501 (2.591 - 2.501)
Space group	P 21 21 2
Unit cell	142.302 316.361 65.647 90 90 90
Total reflections	1245037 (137275)
Unique reflections	92245 (10156)
Multiplicity	13.5 (13.5)
Completeness (%)	88.98 (99.30)
Mean I/sigma(I)	20.01 (2.77)
Wilson B-factor	45.8
R-merge	0.09469 (0.7708)
R-meas	0.09841 (0.8006)
R-pim	0.02649 (0.2143)
CC1/2	0.999 (0.922)
CC*	1 (0.98)
Reflections used in refinement	92171 (10149)
Reflections used for R-free	4607 (510)
R-work	0.1826 (0.2730)
R-free	0.2291 (0.3637)
CC(work)	0.967 (0.921)
CC(free)	0.944 (0.757)
Number of non-hydrogen atoms	19066
macromolecules	18526
ligands	313
solvent	227
Protein residues	2375
RMS(bonds)	0.009
RMS(angles)	1.05
Ramachandran favored (%)	97.8
Ramachandran allowed (%)	2.12
Ramachandran outliers (%)	0.08
Rotamer outliers (%)	1.51
Clashscore	3.97
Average B-factor	45.87
macromolecules	45.97
ligands	45.22
solvent	38.45
PDB deposition:	8ACS
